# A high-density linkage map reveals broad- and fine-scale sex differences in recombination in the hihi (stitchbird; *Notiomystis cincta*)

**DOI:** 10.1038/s41437-024-00711-3

**Published:** 2024-08-02

**Authors:** Hui Zhen Tan, Phoebe Scherer, Katarina C. Stuart, Sarah Bailey, Kate D. Lee, Patricia Brekke, John G. Ewen, Annabel Whibley, Anna W. Santure

**Affiliations:** 1 School of Biological Sciences, University of Auckland, Auckland, New Zealand; 2 Centre for Biodiversity and Biosecurity (CBB), School of Biological Sciences, University of Auckland, Auckland, New Zealand; 3 Institute of Zoology, Zoological Society of London, London, UK; 4 Bragato Research Institute, Lincoln, New Zealand

**Keywords:** Evolutionary biology, Genetic linkage study

## Abstract

Recombination, the process of DNA exchange between homologous chromosomes during meiosis, plays a major role in genomic diversity and evolutionary change. Variation in recombination rate is widespread despite recombination often being essential for progression of meiosis. One such variation is heterochiasmy, where recombination rates differ between sexes. Heterochiasmy has been observed across broad taxonomic groups, yet it remains an evolutionary enigma. We used Lep-MAP3, a pedigree-based software that is efficient in handling large datasets, to generate linkage maps for the hihi or stitchbird (*Notiomystis cincta)*, utilising information from >36 K SNPs and 36 families. We constructed 29 linkage maps, including for the previously unscaffolded Z chromosome. The hihi is an endangered passerine endemic to Aotearoa New Zealand that is sexually dimorphic and exhibits high levels of sexual conflict, including sperm competition. Patterns in recombination in the hihi are consistent with those in other birds, including higher recombination rates in micro-chromosomes. Heterochiasmy in the hihi is male-biased, in line with predictions of the Haldane-Huxley rule, with the male linkage map being 15% longer. Micro-chromosomes exhibit heterochiasmy to a greater extent, contrary to that reported in other birds. At the intra-chromosomal level, heterochiasmy is higher nearer to chromosome ends and in gene-rich regions. Regions of extreme heterochiasmy are enriched for genes implicated in cell structure. This study adds an important contribution in assessing evolutionary theories of heterochiasmy and provides a framework for future studies investigating fine-scale heterochiasmy.

## Introduction

Understanding a species’ adaptive potential via assessing the mechanisms of evolutionary change and genomic variation provides key information for conservation assessments (Steiner et al. 2013). Recombination, which refers to the exchange of DNA material between homologous chromosomes during meiosis, is increasingly recognised as a driver of evolutionary change as it reassorts genetic variation to create novel combinations of alleles that can be inherited by offspring (Posada et al. 2002; Peñalba and Wolf 2020). Recombination is fascinating because whether its effect is beneficial, deleterious, or neutral depends on the combination of alleles that were broken up or formed (Stapley et al. 2017a). The effects of recombination operate at multiple scales within a genome (Myers et al. 2006), interact with selection and gene flow, and have profound implications on genetic diversity (Lercher and Hurst 2002; Ellegren and Galtier 2016; Wong and Filatov 2023), population diversification, and speciation (Ortiz-Barrientos et al. 2016; Samuk et al. 2017). Low recombination rates may reduce the effectiveness of selection since linkage disequilibrium generated from drift is not broken down. Low recombination rates have been associated with an increase in mutation load (Felsenstein 1974) while high recombination rates can increase opportunities for selection and mediate a species’ adaptive potential (Ritz et al. 2017).

Although recombination is usually essential for accurate segregation of chromosomes for meiosis progression and should theoretically be tightly regulated, variation in recombination is a widespread phenomenon (Stapley et al. 2017b). Recombination can vary within and between populations or species, and across the genome (Stapley et al. 2017b). Intriguingly, recombination can also differ between sexes, where recombination may be absent in one sex (achiasmy), or where both sexes are recombining but at different rates (heterochiasmy). Achiasmy is thought to occur as a consequence of recombination suppression in the heterogametic sex having pleiotropic effects across the genome (Haldane-Huxley rule; Haldane 1922; Huxley 1928). It is most often observed in arthropods (Satomura et al. 2019) with the absence of recombination always observed in the heterogametic sex (Burt et al. 1991). Heterochiasmy, a term only recently coined (Lenormand 2003), occurs more commonly and is taxonomically widespread, but less well understood (Morgan 1912, 1914; Burt et al. 1991; Lenormand and Dutheil 2005; Sardell and Kirkpatrick 2020). Heterochiasmy can present itself where one sex is recombining much more than the other (Brelsford et al. 2016), or where sexes differ in the distribution of recombination along the genome (Brelsford et al. 2016; Bergero et al. 2019; Edvardsen et al. 2022). A recent review by Sardell and Kirkpatrick (2020) that included 51 eukaryote species with various sex determination systems revealed broadscale patterns in heterochiasmy, with male recombination often clustered near telomeres, female recombination more evenly distributed or elevated near the centromeres and with overall higher recombination rate in females (Trivers 1988; Brandvain and Coop 2012; Sardell and Kirkpatrick 2020).

Understanding the evolutionary basis of heterochiasmy is potentially significant yet has remained an evolutionary enigma (Lenormand et al. 2016) that does not seem to have a phylogenetic basis (Malinovskaya et al. 2020). Multiple hypotheses (Box 1) have been put forward to explain heterochiasmy, although many may only be applicable to certain species (Dunn and Bennett 1967; Lenormand 2003; Mank 2009; Sardell and Kirkpatrick 2020). Examples of these hypotheses include sexual selection at the diploid (Trivers 1988; Mank 2009) and haploid levels (Lenormand 2003; Lenormand and Dutheil 2005) and sexual dimorphism in the process and control of meiosis. Other explanations involve the action of specific loci which modulate recombination in one sex, thereby favouring heterochiasmy (Petkov et al. 2007; Kong et al. 2014; Ma et al. 2015; Johnston et al. 2016). It has also been proposed that heterochiasmy is an outcome of pleiotropic effects to suppress recombination between non-homologous sex chromosomes (Haldane 1922; Huxley 1928), or is simply neutral variation around the mean recombination rate (Burt et al. 1991).

The availability of large genomic datasets offers a promising opportunity to further characterise heterochiasmy and the conditions that favour its evolution. The need for a pedigree to detect sex differences in recombination can be challenging for wild populations. Despite this, and enabled by software developments (Liu et al. 2014; Rastas 2017; Zheng et al. 2019), high-density linkage maps have been constructed for an increasing number of wild systems, allowing inference of recombination and heterochiasmy landscapes (Johnston et al. 2016, 2017; Dufresnes et al. 2021; Akopyan et al. 2022; McAuley et al. 2024; Bascón-Cardozo et al. 2024). Birds are one of the most well studied taxonomic groups, with comprehensive genomic datasets available (Stiller and Zhang 2019), along with many individual-based monitoring studies that enable pedigree reconstruction (e.g., SPI-Birds Network and Database, Culina et al. 2021). Birds also exhibit a range of life history strategies and high genomic conservation that allow more direct comparisons to improve our understanding of factors that influence heterochiasmy (Malinovskaya et al. 2020; McAuley et al. 2024). Most birds studied to-date do not exhibit pronounced heterochiasmy at the whole genome-level, but the few studies that have characterised fine-scale (i.e., within-chromosome) heterochiasmy have detected localised differences in male and female recombination rates (Groenen et al. 2009; Mank 2009; Kawakami et al. 2014; van Oers et al. 2014; Hagen et al. 2020; Peñalba et al. 2020; Robledo-Ruiz et al. 2022; McAuley et al. 2024). Hence, understanding the genomic drivers of fine-scale heterochiasmy will enable further insights into the evolutionary mechanisms that create and maintain differences in recombination between the sexes.

**Fig. 1:**
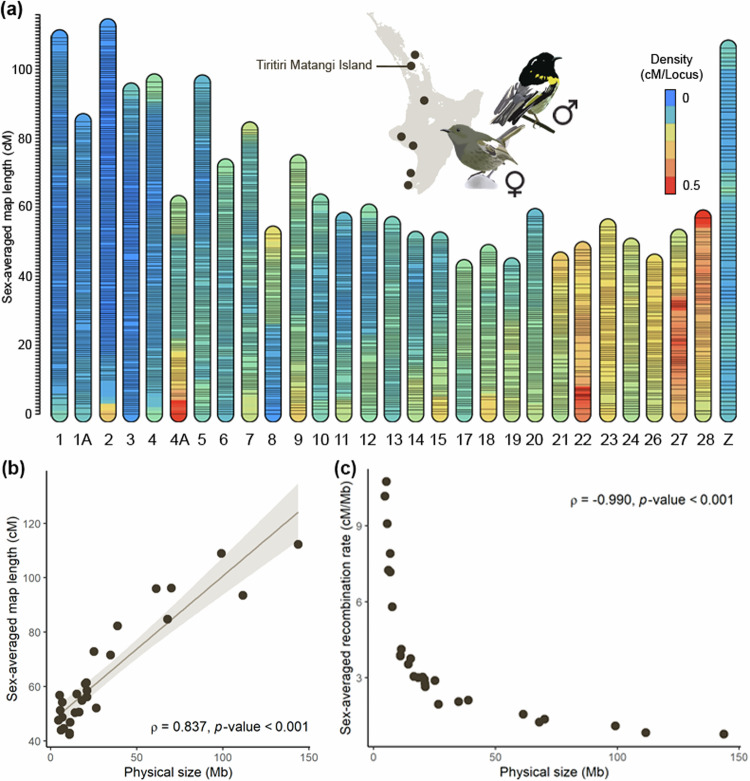
Linkage maps for 29 chromosomes of the hihi constructed from 36 families and 36,304 markers. **a** Linkage maps of the hihi (each vertical bar represents one linkage map) with horizontal lines representing the location of each marker. The background colour of the bars represents local recombination rate and is calculated from centiMorgan (cM) per SNP locus based on windows of 30 SNPs. Given that markers are relatively evenly spread across the genome, cool regions indicate low local recombination rates while hot regions indicate high local recombination rates. The inset map shows the location of present-day hihi populations (dark circles) on the North Island of Aotearoa New Zealand and the label indicates the location of our study population from Tiritiri Matangi Island, accompanied by illustrations highlighting the sexual dimorphism in plumage between male and female hihi (illustrations by Hui Zhen Tan). **b** Comparison of sex-averaged map length (cM) against physical size (Mb) of each autosomal linkage group. Solid brown line represents the linear regression and shaded area represents the 95% confidence interval. **c** Sex-averaged recombination rate (cM/Mb) of each autosomal linkage group against physical size (Mb).

Our study system is the hihi, or stitchbird *Notiomystis cincta*, a passerine endemic to Aotearoa New Zealand. Hihi are sexually dimorphic (Fig. 1) and exhibit high levels of sexual conflict, including a polygynous mating system with forced face-to-face copulations, sperm competition, and some of the highest rates of extra-pair paternity reported in birds (Castro et al. 1996; Ewen et al. 2004; Low 2005, 2006; Brekke et al. 2013) and so – under some theories for heterochiasmy – may have evolved sex differences in recombination. Although once abundant across the North Island, hihi were relegated to a single offshore island following habitat clearance and the introduction of mammalian predators (Taylor et al. 2005). Beginning in the 1980s, a number of hihi populations have been successfully reintroduced, including on the island of Tiritiri Matangi (36°36′8″S, 174°53′13″E) in 1995 (Miskelly and Powlesland 2013). Since establishment, all birds on the island have been individually monitored, including taking blood samples from all nestlings since 2004 (Brekke et al. 2015; de Villemereuil et al. 2019; Duntsch et al. 2020). Hihi are a threatened, taonga (precious) species, and characterisation of their recombination landscape will enable more refined predictions of their limited capacity to adapt to future change (de Villemereuil et al. 2019; Duntsch et al. 2020).

In this study, we leverage existing genomic data and a genetically-verified pedigree for hihi sampled from Tiritiri Matangi (Brekke et al. 2013; Scherer 2017; Duntsch et al. 2022; Lee et al. 2022; Bailey et al. 2023) to construct linkage maps and characterise the landscape of recombination in the hihi. We construct sex-specific linkage maps to explore heterochiasmy at the chromosome level and at finer scales, and test for the relationship between heterochiasmy and gene density as well as distance to nearest chromosome end. Finally, we test whether heterochiasmy is likely to have functional implications by assessing whether there is an enrichment of gene ontology terms in regions of extreme heterochiasmy. Overall, our linkage map adds an important empirical dataset to enable assessment of the hypotheses for the evolution of heterochiasmy.

Box 1 Theories explaining trends in heterochiasmy

**Table:** 

Author	Theory	Prediction
Petkov et al., 2007	**Crossover interference:** Crossover interference creates sex differences in the number of crossover events within chromosomes through chromosomal organisation mechanisms.	The sex with the shorter crossover interference distance (usually females) will have higher recombination rates.
Haldane, 1922; Huxley, 1928	**Haldane-Huxley rule:** Selection against recombination of non-homologous sex chromosomes has pleiotropic effects on genome-wide suppression of recombination in the heterogametic sex.	The heterogametic sex (e.g. XY, ZW) will have much lower or absent recombination.
Lenormand, 2003; Lenormand & Dutheil, 2005	**Haploid selection:** Differential selection on gametes during the haploid stage result in sex-specific recombination rates to preserve favourable allele combinations.	The sex under stronger haploid selection will have reduced recombination.
Many studies e.g. Johnston et al., 2016; Kong et al., 2014; Ma et al., 2015	**Loci-specific:** Heterochiasmy is a result of the action of specific loci affecting recombination in one sex but not in the other, and many such loci have been identified, though most studies are on model animals.	Outcome varies across loci.
Lamb, Sherman, et al., 2005; Lamb, Yu, et al., 2005	**Maternal ageing:** Elevated female recombination rates provide more physical connections between chromosomes to facilitate proper segregation of homologous chromosomes after degradation by time while in meiotic pachytene arrest.	In species with arrested meiosis, female recombination rates will be elevated.
Brandvain & Coop, 2012	**Meiotic drive:** Given that meiosis and its genetic control differs between sexes, sex-specific recombination rates may evolve to modify the efficacy of meiotic drivers.	Females recombine more often near centromeres than do males to constrain meiotic drivers, while males recombine relatively more near telomeres.
Burt et al., 1991	**Neutrality:** Selection acts on the mean value of recombination within a population, and sex-specific differences are caused by variation around an equilibrium, where male and female recombination rates are correlated.	Heterochiasmy represents neutral variation in recombination around a mean.
Trivers, 1988	**Sexually antagonistic selection (1):** Antagonistic selection betweeen sexes results in variance in reproductive success hence recombination is suppressed to preserve beneficial allele combinations.	The sex under stronger sexual selection, usually males, will have reduced recombination.
Mank, 2009	**Sexually antagonistic selection (2):** Changing environments and selection pressures during male-biased dispersal may select for increased recombination in males.	In eutherian mammals with high levels of male-biased dispersal, recombination is higher in males.

## Materials and methods

### Sampling and genotyping

In this study, we utilise data for Tiritiri Matangi individuals from a previously published genetic dataset of the hihi (Lee et al. 2022) which genotyped samples from five different hihi populations on an Affymetrix 50 K single nucleotide polymorphism (SNP) array developed for the hihi. A total of 45,553 SNPs were successfully genotyped (designated as polymorphic and of high resolution according to the default quality control metrics in the Axiom Analysis Suite) on the SNP array (Lee et al. 2022). After genotyping, sample IDs for Tiritiri Matangi individuals were validated using a comprehensive sample verification framework (Duntsch et al. 2022) to yield a final dataset of 483 individuals.

### Full-sibling family pedigrees

The long-term monitoring data from Tiritiri Matangi alongside a microsatellite genotype database was used to infer parentage of all nestlings over the generations (Brekke et al. 2013; Duntsch et al. 2022). To select which of the 483 individuals to utilise in linkage map construction, we first identified every unique pair of SNP array-genotyped parents and built full-sibling families around them with grandparents included if also genotyped. In general, we only retained families with at least two offspring, but also allowed the inclusion of two families that had only one offspring as all grandparents were genotyped for these families. Our final linkage map pedigree consisted of 36 full-sibling families comprising 144 unique individuals. A custom script was used to prepare our pedigree file for input into Lep-MAP3 (see Data availability).

### SNP filtering

SNPs on the SNP array and their flanking probe sequences were mapped to the high quality, contiguous hihi female reference genome assembly (Bailey et al. 2023) using BWA-MEM (Li and Durbin 2009). Of the 45,553 SNPs, 45,369 were assigned a contig position in the hihi assembly. These were subsequently categorised as placed or unplaced, depending on whether they were mapped within contigs that met the size criteria (>50 kb) to be included in the scaffolded hihi genome assembly, which identified 32 autosomal chromosomes along with classifying unscaffolded sex-linked contigs. For placed SNPs, chromosome assignments and base-pair positions were inferred according to their position within the scaffolded hihi genome assembly. For unplaced SNPs, a putative hihi chromosome assignment was inferred based on mapping these smaller contigs against the zebra finch genome (GCA_003957565.4) using RAGTAG v2.1.0 (Alonge et al. 2019) since both genomes are highly syntenic (Bailey et al. 2023) (Fig. S1). SNPs with a RAGTAG confidence score of >0.95 were assigned to chromosome “N_unplaced”, “N” being the putative chromosome assigned, but retain their base pair positions within their respective contigs. All genotyped SNPs were filtered using PLINK v1.9 (Chang et al. 2015) to remove samples exceeding 10% missingness (--mind 0.1) and SNPs exceeding 10% missingness (--geno 0.1) or with minimum minor allele frequency lower than 5% (--maf 0.05). We then extracted autosomal SNPs for further filtering to remove SNPs that deviate significantly from Hardy-Weinberg equilibrium (--hwe 0.05) and that have high Mendelian error rates (--me 1 0.1, with the former value denoting the threshold for per-trio error rate and the latter denoting that for per-variant error rate). SNPs assigned to sex chromosomes were excluded from this filtering step as they would be wrongly flagged under an autosomal model of inheritance. Autosomal SNPs and sex-linked SNPs were then merged back into one dataset. Duplicated SNPs with the same chromosome assignment and base-pair position were present in our dataset due to the inclusion of both forward and reverse probes in the design of the SNP chip. For each pair of duplicated SNPs, the SNP with the lower missingness was retained, and if there was no difference in missingness then the second SNP in each pair was retained. Our final genotype data consisted of 37,607 SNPs, including 1142 which were unplaced.

### Linkage mapping

#### Linkage mapping using Lep-MAP3

We constructed linkage maps for the hihi using Lep-MAP3 which can handle datasets with a large number of SNPs and families (Rastas 2017). First, we used the ParentCall2 module to call possible missing or erroneous genotypes by considering half-sibling information (halfSibs = 1). ParentCall2 was also used to identify sex (Z)-linked SNPs (ZLimit = 1) as an independent check of SNPs assigned to the Z chromosome based on our genome assembly. Genotypes were called if their likelihood was 100x higher than the second-best genotype combination. SNPs were discarded if all parents are either missing or homozygous (removeNonInformative = 1). We skipped the Filtering2 module as we had already filtered for missingness and deviation from Mendelian proportions (segregation distortion) using PLINK. Next, we used the SeparateChromosomes2 module to cluster SNPs into linkage groups. We tested multiple LOD score thresholds (lodLimit = 10–17) and recombination fraction values (theta = 0.01–0.05) but were unable to produce linkage groups representative of chromosomes in the hihi (Fig. S2). Since we have a chromosome-level assembly and are confident of the chromosome assignment of SNPs, we decided to run SeparateChromosomes2 on each chromosome separately as a filtering step rather than for identification of linkage groups. To do so, we split the ParentCall2 output file by chromosome, with unplaced SNPs included with their respective putative chromosomes. SeparateChromosomes2 was then performed on each chromosome using a LOD score limit of 5 (lodLimit = 5) and a fixed recombination fraction of 0.03 (theta = 0.03). This process ensured that SNPs assigned to the same chromosome had correspondingly high likelihoods of linkage, and to exclude SNPs that showed otherwise. Only SNPs assigned to the largest linkage group of each chromosome were retained, which was the large majority of SNPs assigned to that chromosome based on our genome assembly, and were merged into a single file for downstream steps. Finally, we used the OrderMarkers2 module to infer SNP order within each linkage group. For each linkage group, 10 independent runs of OrderMarkers2 were performed using 24 merge (numMergeIterations = 24) and eight polish (numPolishIterations = 8) iterations and the default mapping function (Haldane). For each run, pairwise LOD scores (computeLODScores), position intervals (calculateIntervals) and sex-averaged positions (sexAveraged = 1) of each SNP were calculated. We applied scaling for the seven largest chromosomes to account for an excess number of SNPs in macro-chromosomes relative to the number of individuals sampled (scale = 3M/N 3, where M = number of individuals, N = number of markers; the former value “3M/N” denotes the scaling applied to the genotype likelihood of all markers and the combination of both values “3M/N” and “3” denotes the respective scale factors for the first and last seven possible recombination intervals at the map ends; see Rastas (2023) for a detailed explanation). Information about the number of SNPs retained per chromosome after bioinformatic steps in PLINK and in each module of Lep-MAP3 is provided in Fig. S3.

We also produced a physical map for each chromosome where the order of SNPs was fixed according to their known base-pair position. We did so first for autosomal chromosomes by only using placed SNPs from the ParentCall2 output above and skipped SeparateChromosomes2. A single run of OrderMarkers2 was performed for each chromosome to calculate sex-specific and sex-averaged map positions of each SNP.

A modified approach was taken to construct the linkage map of the Z chromosome which consists of Z-linked contigs that were previously not scaffolded (Bailey et al. 2023). We constructed a priori (i.e., not informed by physical order) linkage maps of the Z chromosome with OrderMarkers2 (as described above for the autosomes) using all Z-linked contig SNPs identified in both the female (reference) genome assembly and also in the male assembly, which was constructed alongside our reference genome (Bailey et al. 2023; Fig. S4). We verified that for both the female and male Z-linked contigs, each contig spanned a unique stretch of the genetic map, suggesting that these Z-linked contigs could be ordered and assembled using the linkage map. We removed three contigs with <3 SNPs and ordered and assembled all other female reference Z-linked contigs using agptools (WarrenLab 2022). Physical positions of the SNPs were updated accordingly and results from the a priori map were visualised against physical position to check that there were no discrepancies. A physical map of the Z chromosome was then constructed using the updated physical positions.

#### Linkage map evaluation

All a priori and physical linkage maps were evaluated using the LMPlot module in Lep-MAP3 which makes a Lep-MAP graph to detect mapping errors. Further checks were also done to detect discrepancies between map and physical positions, by considering map positions against positions in physical maps (only for a priori maps), pairwise LOD score, position intervals, and zebra finch marker order (Rhie et al. 2021; Lee et al. 2022). For a priori maps, the map with the highest likelihood score and with no major errors in the Lep-MAP graph or discrepancies against physical positions was chosen as the final map for each chromosome. Lep-MAP graphs of the maps of chromosome 10 and 29 showed an elevated number of recombinations, across a short span of physical position, at the map ends which indicates phasing or mapping errors. These maps were truncated from the ends to remove the erroneous map positions which resulted in the removal of four and 13 markers from the maps of chromosome 10 and 29 respectively. Linkage maps were also compared to previous maps (Scherer 2017; Bailey et al. 2023) that were constructed using CRI-MAP v2.4 (Green et al. 1990), modified by Xuelu Liu (Mosanto) and CRI-MAP v2.507 (Evans and Maddox 2015). Linkage maps informed by the physical base-pair position of SNPs provided better resolution of recombination landscapes and were chosen for downstream analyses (see Results). Only maps with >70 SNPs were retained to ensure sufficient density of SNPs across the linkage map, hence we obtained fewer autosomal linkage maps than had been annotated in the hihi genome assembly (32 pairs of autosomes; Bailey et al. 2023).

#### Linkage map summary and visualisation

Linkage maps were visualised in LinkageMapView (Ouellette et al. 2018) to display SNP position and density. To visualise linkage map landscapes across the hihi genome and allow comparison across different maps, we created Marey map representations of each linkage group by visualising genetic map distance against physical distance using the ggplot2 package in R 4.2.1 (Wickham 2016; R Core Team 2022). LOESS smoothing was applied to improve visualisation of trends along the chromosome, using an appropriate span parameter for each chromosome that corresponds to roughly 50 SNPs.

### Recombination rate calculation and regression models

Recombination rates for each linkage group were calculated by dividing their respective map length (cM) by physical size (Mb). We calculated the mean autosomal recombination rate of the hihi from the total map length and total physical size of all autosomal linkage groups. It is important to note that in birds, where males are the homogametic sex (ZZ) and females are the heterogametic sex (ZW), recombination between sex chromosomes of females is restricted to small pseudo-autosomal regions (PARs) and creates a situation of extreme heterochiasmy when sex chromosomes are compared. As such, and because the PAR in hihi is not resolved between the Z and W chromosomes (Bailey et al. 2023), calculations of sex-averaged map length and comparisons of heterochiasmy were only done for autosomal chromosomes. The sex-averaged map length and recombination rate of each linkage group were visualised against physical size and correlations were tested using Spearman’s Rho with the corr_test function in the statsExpressions R package (Patil 2021).

We then investigated fine-scale patterns in recombination rate within autosomal chromosomes only. We first defined non-overlapping intervals of 1 Mb along each chromosome and calculated the sex-averaged recombination rate for each of these intervals in R. Recombination rate for an interval was calculated as the genetic distance (cM) between the first and last SNP in the interval divided by the physical distance (Mb) between them. Using available genome annotations for the hihi (Bailey et al. 2023), we then extracted a list of genes that are completely within each 1 Mb interval with the foverlaps function in R (type = ”within”). We then filtered the list of genes in R to remove annotations with unknown gene names. We also calculated the actual and relative distance of the midpoint of each interval to the nearest chromosome end. The actual distance is calculated in bp while the relative distance was standardised from 0 to 0.5, with 0 corresponding to chromosome ends and 0.5 corresponding to chromosome centre in the absence of centromeric information. We visualised the distribution of recombination rate across all intervals between chromosome types (macro- or micro-chromosome; here macro-chromosomes are defined as chromosomes 1–7 & 1A) with the ggplot2 package in R. In addition, we visualised associations between recombination rate with gene density and distance to nearest chromosome end using ggplot2. We also performed regressions to test the strength of these associations using Spearman’s Rho with the corr_test function in the statsExpressions R package.

### Heterochiasmy calculation and regression models

In addition to the sex-averaged map, Lep-MAP3 also infers male- and female-specific linkage maps that allow us to understand the landscape of heterochiasmy. The magnitude and direction of heterochiasmy was quantified globally and per chromosome using the heterochiasmy index:

$$\frac{{female\; map\; length}\left({cM}\right)-{male\; map\; length}({cM})}{{average\; map\; length}({cM})}$$

(Malinovskaya et al. 2020). We also visualised male versus female map lengths for the other high-density avian linkage maps constructed using Lep-MAP3, namely that of the helmeted honeyeater (*Lichenostomus melanops cassidix*; Robledo-Ruiz et al. 2022) and superb fairy-wren (*Malurus cyaneus*; Peñalba et al. 2020).

#### Quantifying heterochiasmy at fine-scales

Marey maps were also used to visualise intra-chromosome heterochiasmy by plotting male- and female-specific genetic map distance against physical distance using the ggplot2 package in R. We opted to use proportional instead of raw change in genetic map distance to improve visualisation of sex differences in the local recombination landscape (i.e., proportional genetic map distances vary from 0 to 1 for both males and females). LOESS smoothing was applied to visualise trends in heterochiasmy along the chromosome, using an appropriate span parameter for each chromosome that corresponds to roughly 50 SNPs.

To investigate fine-scale heterochiasmy, we calculated sex-specific recombination rates and, from this, the heterochiasmy index for each 1 Mb interval in R. When both sexes are recombining at the same rate within an interval, the heterochiasmy index will be 0. In contrast, when neither sex recombines (and sex-averaged change in map distance is 0 cM), the heterochiasmy index will have an undefined value. For the latter scenario, we also gave the heterochiasmy index a value of 0 since there are no sex differences in recombination. The heterochiasmy index has a maximum value of 2 (no recombination in males) and a minimum value of −2 (no recombination in females). Other than heterochiasmy index, which is a measure of relative difference in recombination between sexes, we also calculated the difference in recombination rates between sexes $({female\; recombination\; rate}-{male\; recombination\; rate})$. For both heterochiasmy index and difference in recombination rates between sexes, negative values reflect higher recombination in males while positive values reflect higher recombination in females. As we had done for recombination rate, we visualised the distribution of values of the difference in recombination rate between sexes and heterochiasmy index for each 1 Mb interval between macro- and micro-chromosomes with ggplot2. In addition, we visualised associations between heterochiasmy with gene density and distance to nearest chromosome end using ggplot2 and performed regressions to test the strength of these associations. For regressions using the heterochiasmy index as our measure of heterochiasmy, we first took the absolute value of the heterochiasmy index and rescaled it to be bounded by 0 and 1 by dividing the value by 2. This is so that we can infer associations between these genomic features with the extent of heterochiasmy (and not the direction of it). We then applied a zero-or-one inflated beta (zoib) regression model to account for the bounded values using the brmsformula function in the brms R package (Bürkner 2017). For regressions using the difference in recombination rates between sexes, we also took the absolute value and tested these associations using Spearman’s Rho with the corr_test function in the statsExpressions R package.

#### Regions of elevated heterochiasmy and gene overrepresentation tests

To further characterise regions of elevated heterochiasmy, we conducted outlier analyses to identify regions with the largest differences in recombination rates between sexes. We used the difference in recombination rates between sexes as a direct measure of heterochiasmy. We did not use heterochiasmy index, a relative measure of heterochiasmy, as it can identify regions as outliers despite small differences (in raw values) in recombination rates between sexes. Intervals were defined as outliers if they have a value smaller than the lower critical value (male-biased), defined as $(25{th\; percentile}-\left(1.5\times {IQR}\right))$, where IQR refers to the interquartile range, or larger than the upper critical value (female-biased), defined as $(75{th\; percentile}+\left(1.5\times {IQR}\right))$. For gene density, we visualised the distribution of values across all outlier intervals versus non-outlier intervals, between chromosome types and between female- versus male-biased intervals. For relative distance to nearest chromosome end, we visualised the distribution of values across all outlier intervals between female- versus male-biased intervals.

We also identified regions of extreme heterochiasmy which harbour the largest differences in recombination rates between sexes. Intervals were defined as outliers of extreme heterochiasmy if they have a value smaller than the lower critical value (male-biased), defined as $25{th\; percentile}-(3\times {IQR})$ or larger than the upper critical value (female-biased), defined as $75{th\; percentile}+(3\times {IQR})$. We performed gene overrepresentation tests within regions of extreme heterochiasmy using the web tool of PANTHER v17.0 (Mi et al. 2019; Thomas et al. 2022) against a reference list of genes on the chromosome-level assembly of the hihi. We performed the test for biological, molecular, and cellular processes on both the GO (gene ontology) and PANTHER GO-Slim annotation datasets using the Fisher’s exact test with false discovery rate (FDR) correction. GO terms are significantly overrepresented if their *p*-value and FDR are <0.05.

## Results

### Linkage maps

#### Hihi linkage maps constructed by Lep-MAP3

Linkage maps constructed by Lep-MAP3 without a priori marker order information (‘a priori map’) produced spurious breakpoints and showed poor marker order synteny in areas with low or no recombination (Fig. S5) despite increasing the scale parameter to account for an excess of SNPs relative to number of individuals. Therefore, maps constructed based on the physical ordering of markers in the hihi genome assembly (‘physical map’) were chosen as the final maps. Furthermore, physical maps showed very similar recombination landscapes to maps ordered by Lep-MAP3, confirming that the known physical order of SNPs corroborates with linkage information. A total of 29 linkage maps were produced for 29 chromosomes of the hihi including the Z chromosome (Table 1, Fig. 1a). The scaffolded hihi genome (Bailey et al. 2023) annotated 32 autosomal pairs, of these, linkage maps for chromosomes 16, 25A, 25B and 29 were not retained in downstream analyses due to a low number of SNP markers (<70 SNPs) mapped to them. Linkage maps were not constructed for smaller, unscaffolded contigs in the assembly, which are likely to represent further micro-chromosomes (Bailey et al. 2023). The autosomal linkage maps of the hihi have a total length of 1,799.68 cM and comprise 33,890 SNPs (Table 1). The linkage map of the Z chromosome is 106.04 cM in length and comprises 2414 SNPs (Table 1). The sex-averaged linkage map length was significantly and positively associated with the physical size of the chromosome (Fig. 1b).

**Table 1:** Summary of linkage groups in the hihi genome.

Chromosome	Physical size (Mb)	No. of SNPs	Map length (cM)			Heterochiasmy index	Recombination rate (cM/Mb)		
			Sex-averaged	Female	Male		Sex-averaged	Female	Male
1	99.26	3530	108.97	97.5	121.82	−0.22	1.1	0.98	1.23
1A	68.01	2326	84.75	77.69	92.94	−0.18	1.25	1.14	1.37
2	143.7	4897	112.22	97.25	128.89	−0.28	0.78	0.68	0.9
3	111.67	3476	93.56	93.93	94.47	−0.01	0.84	0.84	0.85
4	70.22	2453	96.24	92.13	102.26	−0.11	1.37	1.31	1.46
4A	20.1	684	60.96	50.89	71.9	−0.34	3.03	2.53	3.58
5	61.39	1986	95.94	83.84	109.18	−0.26	1.56	1.37	1.78
6	34.87	1257	71.6	60.69	83.42	−0.32	2.05	1.74	2.39
7	38.89	1259	82.3	77.56	88.06	−0.13	2.12	1.99	2.26
8	26.64	950	52.11	45.28	59.65	−0.28	1.96	1.7	2.24
9	25.15	895	72.8	70.95	75.52	−0.06	2.89	2.82	3
10	20.9	1082	61.41	54.16	69.63	−0.25	2.94	2.59	3.33
11	21.17	1125	56.09	47.28	65.69	−0.33	2.65	2.23	3.1
12	21.11	1089	58.54	44.7	73.05	−0.48	2.77	2.12	3.46
13	18.28	918	54.88	53.96	56.51	−0.05	3	2.95	3.09
14	16.57	846	50.57	40.76	61.04	−0.4	3.05	2.46	3.68
15	14.26	727	50.35	48.9	52.49	−0.07	3.53	3.43	3.68
17	10.98	569	42.38	44.95	40.29	0.11	3.86	4.09	3.67
18	11.36	525	46.82	44.54	49.69	−0.11	4.12	3.92	4.37
19	11.02	527	42.91	42.35	44.13	−0.04	3.89	3.84	4.01
20	15.23	788	57.24	62.55	52.64	0.17	3.76	4.11	3.46
21	7.69	334	44.62	43.53	46.2	−0.06	5.8	5.66	6.01
22	4.68	196	47.57	48.03	47.85	0	10.16	10.26	10.22
23	6.87	322	54.25	51.9	57.41	−0.1	7.9	7.55	8.36
24	6.77	400	48.61	43.16	54.72	−0.24	7.18	6.37	8.08
26	6.07	276	43.97	42.99	45.46	−0.06	7.24	7.08	7.49
27	5.64	215	51.21	60.95	42.34	0.36	9.08	10.8	7.5
28	5.29	238	56.81	58.11	56.64	0.03	10.73	10.98	10.7
**Total (autosomes)**	903.78	33,890	1,799.68	1,680.50	1,943.86	−0.15	-	-	-
**Mean (autosomes)**	31.16	1,210.36	62.06	57.95	67.03	-	1.99	1.86	2.15
Z	73.43	2414	-	-	106.04	-	-	-	1.44
**Total (all)**	977.21	36,304	-	-	-	-	-	-	-

Heterochiasmy index is calculated by (female – male)/(sex-averaged map length) (cM). Mean recombination rate (cM/Mb) is calculated from the total physical size of all chromosomes and total map length of all linkage groups and does not represent the mean of recombination rates (cM/Mb) across all linkage groups.

#### Comparison of linkage maps constructed by Lep-MAP3 and CRI-MAP

The total autosomal length of linkage maps constructed in Lep-MAP3 is shorter than maps made with fewer SNPs, albeit more individuals, using CRI-MAP (Fig. S6). This difference is attributable to the macro-chromosomes, which had much smaller maps in Lep-MAP3 than in CRI-MAP while micro-chromosomes showed less deviation in map lengths between programs. The three largest chromosomes (chromosomes 1, 2, 3) had Lep-MAP3 map lengths that were less than half of that constructed in CRI-MAP (Figs. S6 and S7). Within chromosomes however, both programs were remarkably similar in recombination landscapes across all chromosomes, in terms of changes in genetic distance across the chromosome (Fig. S7). Both programs identified a higher recombination rate in males (Table 1, Scherer 2017).

#### Application of linkage maps

Linkage maps constructed in Lep-MAP3 without a priori genome mapping information revealed, in chromosome 18, a discrepancy in marker order between the genetic map and physical positions (Fig. S8a, b) from the existing genome assembly of the hihi (Bailey et al. 2023). For this chromosome, we identified an inversion in a repeat-rich region that is poorly covered by sequencing reads, indicating assembly error (Fig. S8c). The contig ordering within chromosome 18 was therefore updated in accordance with that in the genetic map, and this new marker order was used to produce the final chromosome 18 linkage map (Table 1, Fig. 1). For the Z chromosome, which was previously unassembled, a priori linkage maps allowed assembly of the contigs according to genetic map positions.

### Recombination rates and associations

**Fig. 2:**
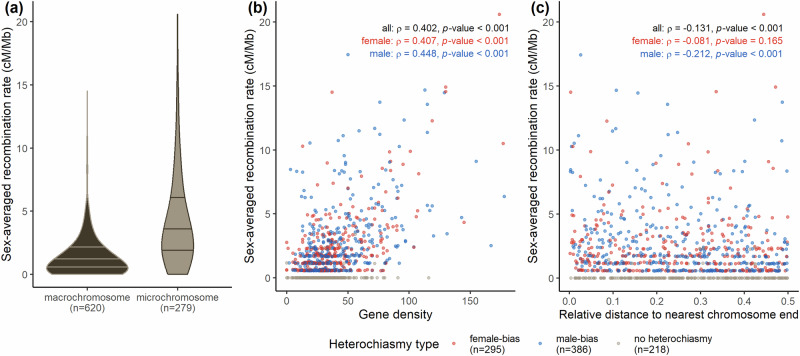
Recombination rate varies within the hihi genome with respect to chromosome type, gene density, and relative distance to nearest chromosome end. **a** Violin plot of sex-averaged recombination rates in cM/Mb across all 1 Mb interval for macro-chromosomes versus micro-chromosomes of the hihi. Horizontal lines on the violin plot (starting from the bottom) represent the 25^th^, 50^th^, and 75^th^ percentile. Relationship between sex-averaged recombination rate (cM/Mb) and (**b**) gene density and (**c**) relative distance to nearest chromosome end for all 1 Mb intervals. Intervals are coloured by whether they have higher female or male recombination rates (i.e., female- or male-biased). Inset in panels (**b**, **c**) are results of Spearman’s Rho correlation tests of sex-averaged recombination rate (cM/Mb) against gene density and relative distance respectively. The number of intervals in each category is provided in parentheses in the legend.

The hihi has a mean autosomal recombination rate of 1.99 cM/Mb, with recombination rates being higher in smaller chromosomes (Table 1, Figs. 1c, 2a). Recombination rate was significantly associated with both gene density (Fig. 2b) and distance to nearest chromosome end (Fig. 2c).

### Heterochiasmy and associations

**Fig. 3:**
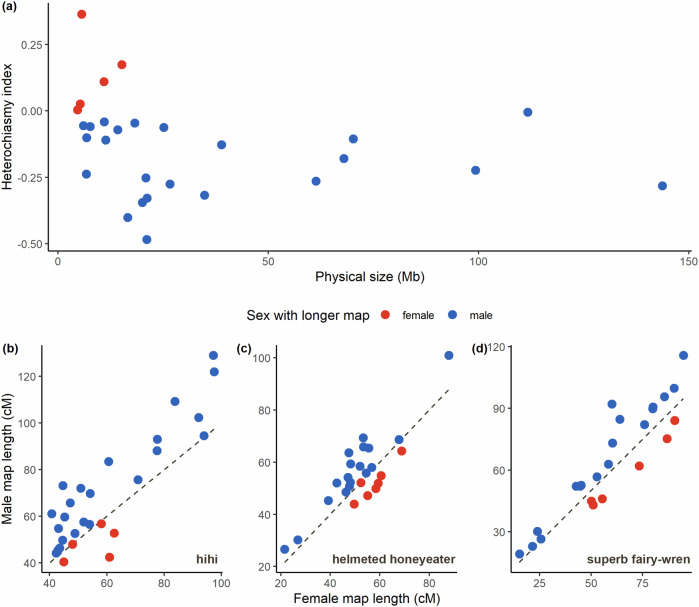
Sex-bias in recombination in available high-density avian linkage maps. **a** Heterochiasmy index plotted against physical size for each of the 28 hihi autosomes. **b**–**d** Comparison of male and female map length of each autosomal linkage group for available high-density avian linkage maps for (**b**) hihi, (**c**) helmeted honeyeater and (**d**) superb fairy-wren. Blue and red point colours represent whether a linkage group has a longer male or female map respectively. Dotted line represents a 1:1 relationship between female and male map length.

We detected differences in sex-specific recombination rates in the hihi, with the autosomal maps being 15.7% longer in males than in females (Table 1). Most chromosomes had longer male maps in the hihi, as is also true for two other passerines with high-density linkage maps constructed with Lep-MAP3: the helmeted honeyeater and superb fairy-wren (Fig. 3; Peñalba et al. 2020; Robledo-Ruiz et al. 2022). In the hihi, only five chromosomes had longer female linkage maps and all five were micro-chromosomes (Table 1; Fig. 3a, b). In the helmeted honeyeater and superb fairy-wren, chromosomes that had longer female maps were of a range of sizes and included macro-chromosomes as well (Fig. 3c, d).

**Fig. 4:**
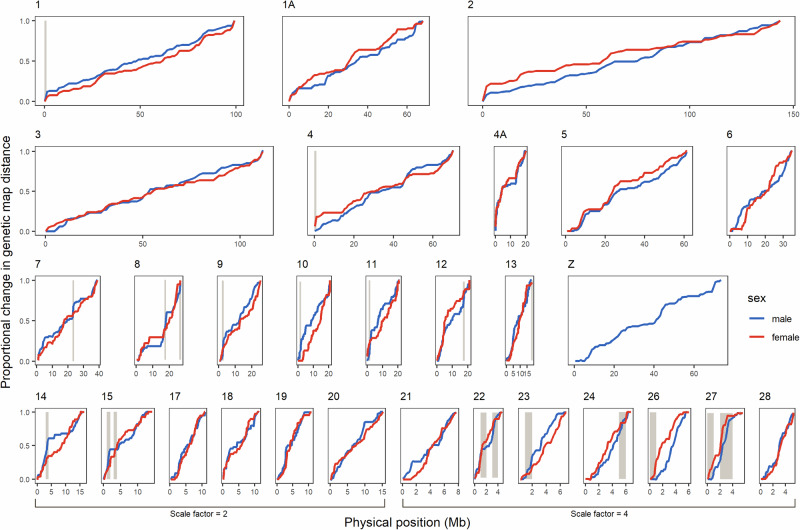
Male and female Marey maps for 29 hihi chromosomes. Maps have been scaled by proportion of the total genetic map length for each sex. Brown shaded boxes represent the intervals of extreme heterochiasmy (largest difference between male and female recombination rates per 1 Mb) that were identified. Widths of the plots for each chromosome are scaled according to physical size, but the x-axes of chromosomes 14–28 are scaled relative to other chromosomes for visualisation purposes. Gradient of the lines in the plot represents recombination rate, with steeper lines denoting high recombination rate.

**Fig. 5:**
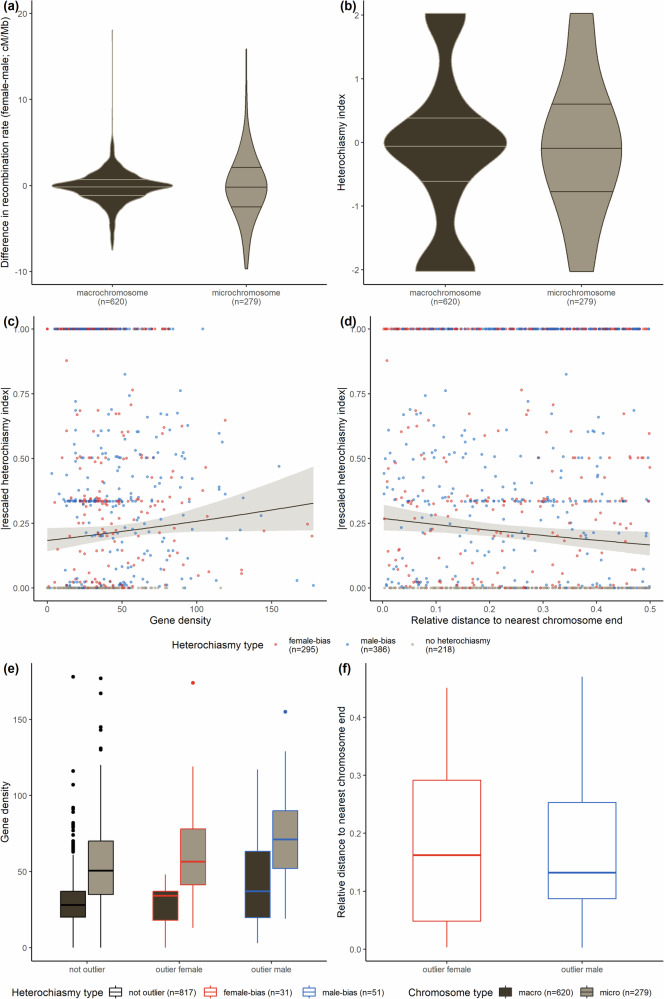
Heterochiasmy varies within the hihi genome with respect to chromosome type, gene density, and relative distance to nearest chromosome end. Violin plots of (**a**) difference in recombination rate between sexes and (**b**) heterochiasmy index across all 1 Mb interval for macro-chromosomes versus micro-chromosomes of the hihi. Horizontal lines on the violin plot (starting from the bottom) represent the 25^th^, 50^th^, and 75^th^ percentile. Graphs of the relationship between the absolute value of the rescaled heterochiasmy index and (**c**) gene density, and (**d**) relative distance to nearest chromosome end for 1 Mb intervals across the genome. Both strongly female-biased and male-biased recombination has a high rescaled absolute value of the heterochiasmy index, while values near zero indicate little to no heterochiasmy. Plots also show the estimated mean and 95% confidence interval modelled from a zero-or-one inflated beta regression (also see Table S1). Boxplots showing the distribution of values of (**e**) gene density and (**f**) relative distance to nearest chromosome end in outlier intervals identified using the difference in recombination rates between sexes. Values across different chromosome types and heterochiasmy types are displayed and the number of intervals in each category is provided in parentheses in the legend.

Heterochiasmy can also be observed within hihi chromosomes at the interval level, but with varying landscapes across chromosomes (Fig. 4). Non-overlapping intervals of 1 Mb in micro-chromosomes exhibit a larger range of heterochiasmy than intervals from macro-chromosomes (Fig. 5a, b). The results of zero-or-one inflated beta regression modelling with the absolute rescaled heterochiasmy index indicate that heterochiasmy has a weak positive association with gene density (the credible interval for the estimate is positive but may include 0; Table S1a, Fig. 5c) and occurs to a greater extent nearer to chromosome ends (credible intervals for the estimate for both raw and relative distance are negative and exclude 0, and Table S1b and c, Fig. 5c, d). Using the raw sex difference in recombination rates however, revealed that heterochiasmy is strongly and positively associated with gene density but strongly and negatively associated with relative distance to chromosome end (Fig. S9).

Both female- and male-biased outlier intervals (i.e., intervals with a substantially higher recombination rate in one sex) have higher gene density than non-outlier intervals (Fig. 5e), while male-biased outlier intervals tend to be nearer to chromosome ends than female-biased outlier intervals (Fig. 5f). Across the genome, 21 intervals of extreme heterochiasmy were identified from outlier analysis, containing 1126 genes (Fig. 4). We found significant overrepresentation of two GO terms (GO:0005882, GO:0045111) in these intervals, both of which are associated with intermediate filament which has functions in cytoskeletal structure (Tables S2 and S3).

## Discussion

In this paper, we show that recombination rate in hihi differs between males and females, and that this heterochiasmy is male-biased. We characterise heterochiasmy in the hihi at intra-chromosomal levels, and show that at fine scales, heterochiasmy is more prevalent in micro-chromosomes and nearer to chromosome ends and in regions of higher gene density. The linkage maps developed for the hihi also enabled us to improve the existing reference genome, including correcting a misassembly on chromosome 18 and inferring contig order within the Z chromosome.

### Linkage map construction methods and utility

#### Physical position of markers informed linkage mapping

In our dataset, the use of marker physical position information improved the linkage maps that were constructed while allowing post-construction validation. Lep-MAP3 was unable to recreate linkage groups corresponding to our known, assembled chromosomes, which we hypothesise is due to the low overall genetic diversity and high levels of inbreeding in the species that has resulted in long-range linkage disequilibrium between markers across chromosomes. Given the limited number of families available to construct our linkage maps, we also found that there was poor resolution of marker order in regions of low recombination when genome order was not provided. Although increasing the scaling parameter in Lep-MAP3 improved the a priori maps of some linkage groups, we preferred to use physical maps as the final maps, especially considering there was an existing high-quality genome for the hihi and that a priori and physical linkage maps showed very similar recombination landscapes (Fig. S10). While the use of an existing genome order precludes discovery of inter-chromosomal rearrangements due to genome assembly error, none of the constructed physical maps showed indications of misassembly such as steep increases in genetic distance. Hence, we opted to proceed with the physical maps which leverage on existing chromosome position information (Bailey et al. 2023).

#### Maps produced by Lep-MAP3 and CRI-MAP are similar in recombination landscape but deviate in lengths

Across the genome, the striking similarity in linkage maps constructed by Lep-MAP3 and CRI-MAP provides somewhat independent validation of the recombination landscape in hihi (Fig. S7), especially given that, because CRI-MAP can incorporate information from more complex family relationships (Green et al. 1990), the 144 Lep-MAP3 individuals were only a subset of those used in CRI-MAP (436 birds). However, we note that recombination landscape similarities between the programs might be due to similarities in the ordering algorithms of the two programs in using maximum likelihood and local reordering of small windows of markers (7 in CRI-MAP, 5 in Lep-MAP3; Rastas 2017; Scherer 2017). Lep-MAP3 performed the replicates of recursive ordering of markers in much less time than CRI-MAP despite having 18 times more markers, making Lep-MAP3 much more tractable for construction of high-density linkage maps (Bilton et al. 2018). We did however find that CRI-MAP maps were generally longer, as has been seen in other studies (e.g., McAuley et al. 2024). Since the deviation in linkage map length between programs seems to scale with the physical size of the chromosomes, this might suggest an accumulation of errors with increasing chromosome size that would have resulted in map length inflation (Fig. S6; Bilton et al. 2018). Differences in map lengths should also be expected when constructed by different programs and future comparative analyses should take such systematic differences into account when comparing across programs.

#### Linkage maps improve existing genome assembly

Linkage maps were applied in revising the existing long-read genome assembly of the hihi (Bailey et al. 2023) at the scaffold-level. De novo assembly of genomes can be challenging due to a lack of ultra-long reads and the presence of repeat content (Sohn and Nam 2018). Here, we have been able to correct an assembly error caused by misassembly of a repetitive region. We have also been able to order contigs within the Z chromosome, which was previously challenging to assemble because the primary genome assembly was of a female, heterogametic individual where sequencing coverage for Z is only half that of autosomes. Linkage maps complement assembly efforts by providing an anchor for chromosome-scale sequences and an independent validation of the assembly (Fierst 2015). Our linkage maps constructed without a priori marker order information (Fig. S10) will also be useful in assembly of small contigs that are currently unplaced in the genome due to low confidence in their chromosomal assignment (Bailey et al. 2023).

### Landscape of recombination in the hihi in accord with present knowledge of avian species

The mean autosomal recombination rate of the hihi (1.99 cM/Mb) is comparable that that of the helmeted honeyeater (1.83 cM/Mb; Robledo-Ruiz et al. 2022) and superb fairy-wren (1.74 cM/Mb; Peñalba et al. 2020), which are the other avian species with high-density linkage maps constructed with Lep-MAP3. Linkage maps of the hihi fit presently known associations with physical size, where larger chromosomes have longer linkage maps (Fig. 1; Kawakami et al. 2014; Peñalba et al. 2020). Further, higher recombination rates are observed for smaller chromosomes (Figs. 1b and 2a; Haenel et al. 2018). This has been attributed to the fact that in many species, physical connections between homologous chromosomes are necessary for proper segregation during meiosis (Whitby 2005), which leads to at least one recombination event, hence increasing the recombination rate of smaller chromosomes (Brandvain and Coop 2012). Their small size and low marker numbers makes the construction of linkage maps challenging for avian micro-chromosomes (e.g., this study; Peñalba et al. 2020; Robledo-Ruiz et al. 2022). However, the very high synteny of gene order within avian genomes means that syntenic chromosomes may be easily identified for further comparative analysis of the landscape of recombination across the genome.

The significant association of recombination rate with gene density (Fig. 2b) and distance to nearest chromosome end (Fig. 2c) has been observed in other birds (Backström et al. 2010; Kawakami et al. 2014; Peñalba et al. 2020). Chromosome 4A displayed an elevated recombination rate at the beginning of the chromosome which may reflect its interesting evolutionary history amongst birds (Fig. 1a; Sigeman et al. 2020; Huang et al. 2022). Visually however, the pattern of elevated recombination towards the end of chromosomes is not as obvious as that in other avian species (Fig. S11; Peñalba et al. 2020; van Oers et al. 2014).

### Choice of measure of heterochiasmy constrains interpretations

There are multiple measures of heterochiasmy that have been used to quantify sex differences in recombination rates (Table S4). For each of these measures, different data describing recombination (and therefore linkage), such as linkage map length (cM), recombination rate (cM/Mb) or crossover count (which could be converted into cM where one crossover = 50 cM) can be used as input. At intra-chromosomal levels, many of these measures would become mathematically undefined if one sex is not recombining, although this does not affect the heterochiasmy index. By applying the difference in recombination and the heterochiasmy index measures in our analyses, we were able to define a value for each 1 Mb interval in the genome, although we do note that this has resulted in a clustering of extreme maximum and minimum values corresponding to no recombination in one sex. Users of any measures of heterochiasmy should be aware of the associated pros and cons for accurate and contextualised interpretations.

### Heterochiasmy in the hihi is male-biased and is more prevalent nearer to chromosome ends

Heterochiasmy in the hihi is male biased, corroborating existing knowledge of recombination in the hihi (Fig. 3; Table 1; Scherer 2017). Relative to other avian linkage maps, the extent of heterochiasmy in the hihi is higher than that in other species also showing male-biased heterochiasmy, sexual dimorphism, or sexual selection (Malinovskaya et al. 2020; Peñalba et al. 2020) and could be a way to resolve sexual conflict in the hihi (van Doorn and Kirkpatrick 2007). Micro-chromosomes seem to be driving variation in heterochiasmy across the hihi genome (Figs. 3 and 5). Micro-chromosomes are known to exhibit higher recombination rates and tend to be more gene-rich (Fig. 2; Bailey et al. 2023; Burt 2002), creating ample opportunities for selection that can result in differences in recombination rates between sexes. This is however not observed in other avian species with high-density linkage maps, where the extent of heterochiasmy is greater in macro-chromosomes (Fig. 3). At fine scales, a greater extent of heterochiasmy is observed nearer to chromosome ends (Figs. 5 and S9), in accordance with the trend of higher male recombination rates nearer to chromosome ends and more evenly distributed female recombination (Figs. 2 and S11; Brandvain and Coop 2012; Sardell and Kirkpatrick 2020; Trivers 1988). Regions of extreme heterochiasmy in the hihi were enriched for two GO terms linked to cytoskeletal structure. Genes with these GO terms have been associated with structural variants in chickens and quails (Wang et al. 2012; Wu et al. 2018). Structural variants are likely to interact with local recombination although it is unclear how that might lead to diverging sex-specific recombination rates (Wellenreuther et al. 2019; Edvardsen et al. 2022).

### Evaluating heterochiasmy in avian systems in the context of evolutionary hypotheses

Our high density linkage map has allowed investigation into both global and fine-scale patterns of heterochiasmy in the hihi. This research provides novel insight into heterochiasmy in avian systems, which is known to be less extreme than for other animal groups (Sardell and Kirkpatrick 2020). Male-biased heterochiasmy in the hihi fits the prediction of the Haldane-Huxley rule where the homogametic sex (males in birds) has a higher recombination rate. In contrast, hihi recombination rates do not seem to be explained by the theory that the sex experiencing the most variable selection pressures would have the highest recombination rates (Mank 2009). Hihi on Tiritiri Matangi (where this study is based) exhibit higher recombination rate in males despite female-biased dispersal (Richardson et al. 2010). It is however important to consider that male-biased dispersal has been observed at another hihi population (Rutschmann et al. 2020) which was founded by individuals from Tiritiri Matangi, and that dispersal in all hihi populations is constrained by the boundaries of their respective sanctuaries. The observed heterochiasmy also does not fit the prediction that the sex under greater selection pressures would have reduced recombination (Trivers 1988), since male hihi are likely to be under greater selection pressures (Walker et al. 2014). A past study on birds similarly found no obvious correlation between heterochiasmy and sexual dimorphism (Malinovskaya et al. 2020). The increase in heterochiasmy in regions enriched for genes suggests that heterochiasmy could have functional implications instead of being a neutral variation (Burt et al. 1991). For other theories, such as haploid selection, meiotic drive, and crossover interference, we would require additional data for interpretation of the observed heterochiasmy. However, considering the differences in male and female meiosis, and the observation of post-copulatory mechanisms that mediate breeding success in the hihi (Brekke et al. 2012), we expect that these theories have potential in explaining heterochiasmy in the hihi.

## Conclusion

Much remains to be understood about sex differences in recombination, or heterochiasmy, despite being widely observed. Our study leverages genomic and life history data of the hihi to characterise heterochiasmy. We found a greater extent of heterochiasmy closer to chromosome ends and with higher gene density. Heterochiasmy in the hihi was not explained by most existing theories of heterochiasmy that we could assess. The high-density linkage map we developed will also be applicable in genomic imputation and in genome-wide association studies. There currently exists a taxonomic gap in avian linkage maps where only four orders (Anseriformes, Galliformes, Columbiformes, Passeriformes) are represented by at least one linkage map (McAuley et al. 2024; Bascón-Cardozo et al. 2024). Further, although increasingly recombination is characterised by population resequencing data (e.g. Bascón-Cardozo et al. 2024), this does not allow for the investigation of sex-specific rates of recombination as is possible from pedigree data. We therefore encourage the creation of sex-specific high density linkage maps, and the sharing of these linkage maps (https://github.com/tanhuizhen/Hihi_Linkage-mapping/tree/main/Results_hihi_linkage_map) for inclusion in meta-analysis to further understand the conditions that lead to the evolution of heterochiasmy.

## Supplementary information

Supplementary material

## Data Availability

Hihi are of cultural significance to the Indigenous People of Aotearoa New Zealand, the Māori and are considered a taonga (treasured) species whose whakapapa (genealogy) is intricately tied to that of Māori. For this reason, the SNP genotypes and associated metadata for hihi will be made available by request on the recommendation of Ngāti Manuhiri, the iwi (extended kinship group) that affiliates as kaitiaki (guardians) for hihi. To obtain contact details for the iwi, please contact Dr Anna Santure: a.santure@auckland.ac.nz. Pipelines, analysis codes and the linkage maps (marker names and locations) are available on GitHub: https://github.com/tanhuizhen/Hihi_Linkage-mapping.
