# Supplementary material for: A high-density linkage map reveals broad- and fine-scale sex differences in recombination in the hihi (stitchbird; Notiomystis cincta)
Source: Heredity (Edinb). 2024 Aug 2;133(4):262–75. doi: 10.1038/s41437-024-00711-3 (PMC11437212; doi:10.1038/s41437-024-00711-3)
Supplement: Supplementary file 1 — Supplementary material [file 41437_2024_711_MOESM1_ESM.pdf]

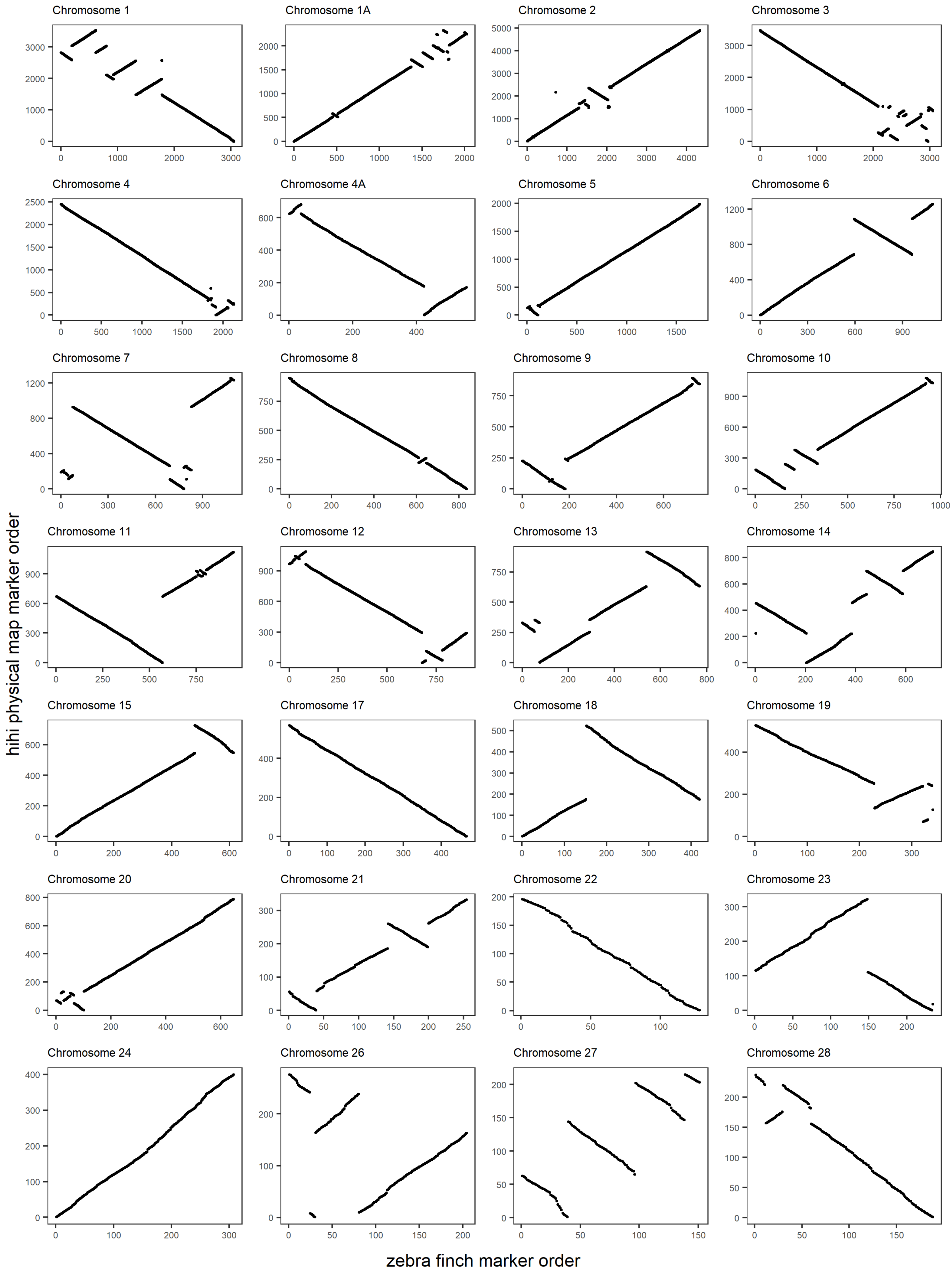

Figure S1. Order of markers in hihi based on the published genome assembly ("hihi physical map") versus the zebra finch marker order. Hihi SNP markers were mapped to the hihi genome using BWA-MEM and to the zebra finch genome using BLAST.

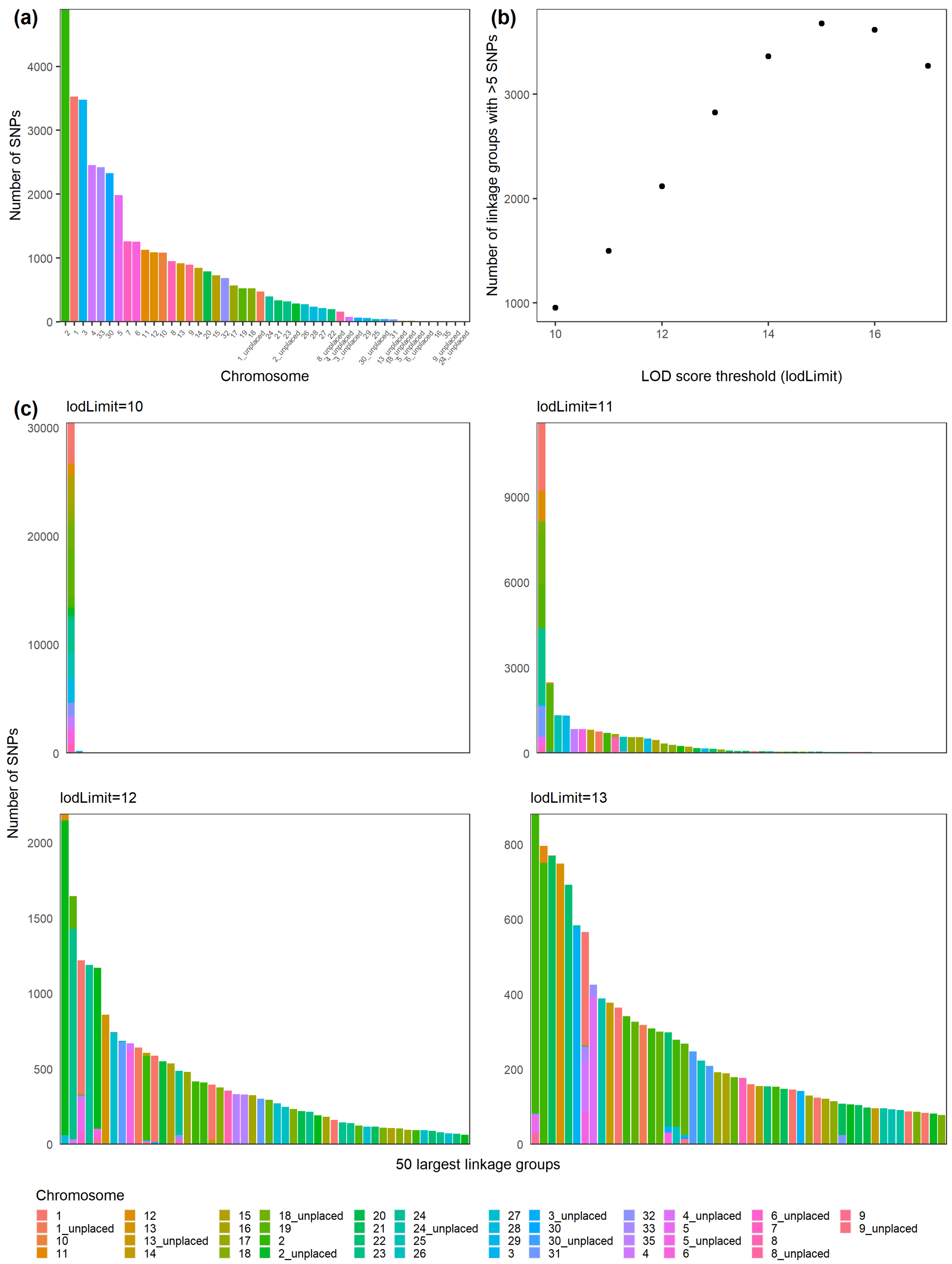

Figure S2. Results of SeparateChromosomes2 using different lodLimits. Only the largest 50 linkage groups are shown in each plot. (a) Number of SNPs aligned to the hihi reference genome for each chromosome; unplaced SNPs are those on contigs that did not meet the size threshold to be included in the genome assembly. (b) Plot showing the number of linkage groups with more than 5 SNPs identified at different LOD score thresholds. (c) Identification of linkage groups from SeparateChromosomes2 across LOD score thresholds 10–13; note that none of these thresholds recreate the known genome structure (panel (a)).

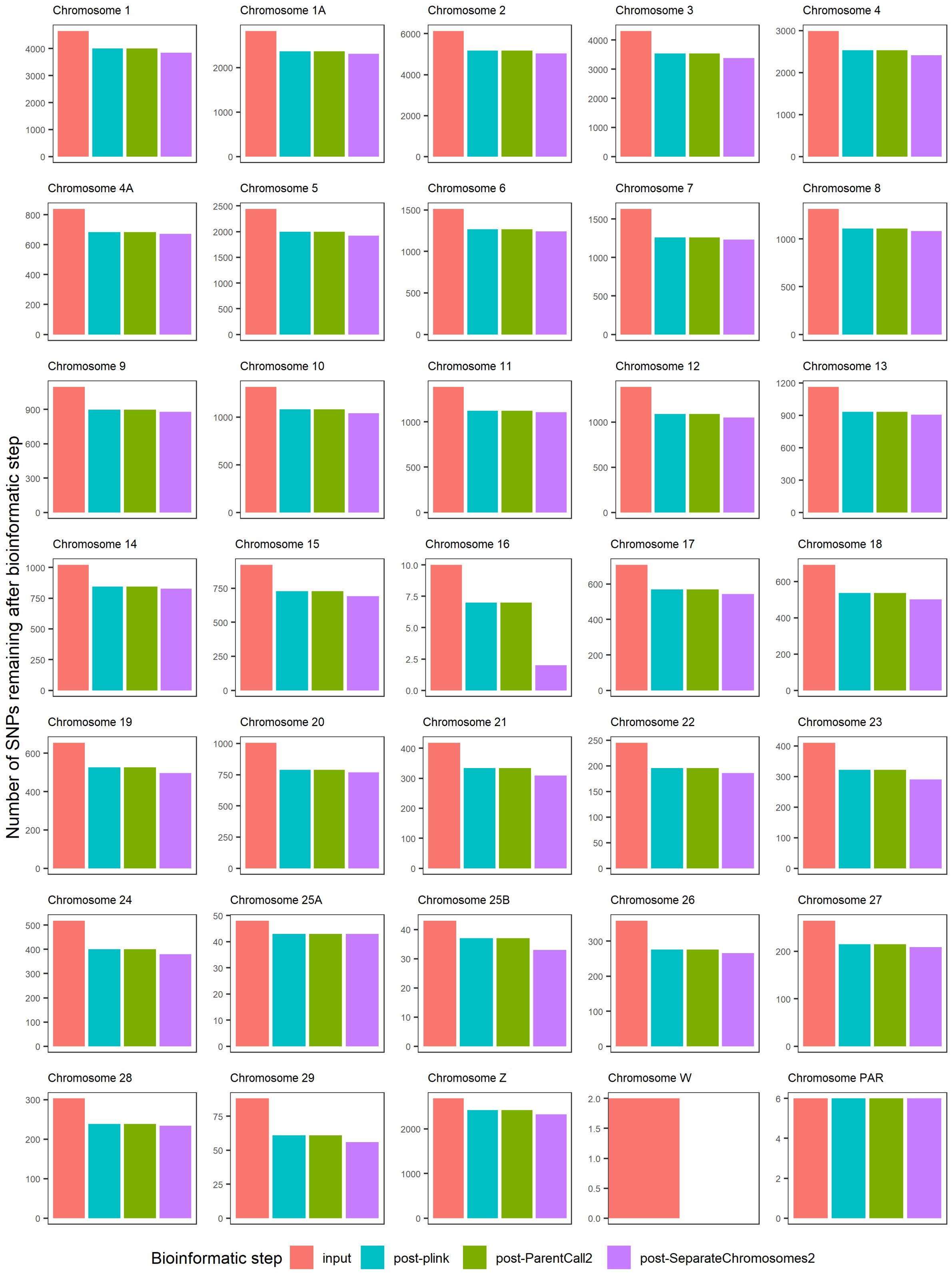

Figure S3. Information about the number of SNPs retained in each chromosome after bioinformatic steps in PLINK and in each module of Lep-MAP3.

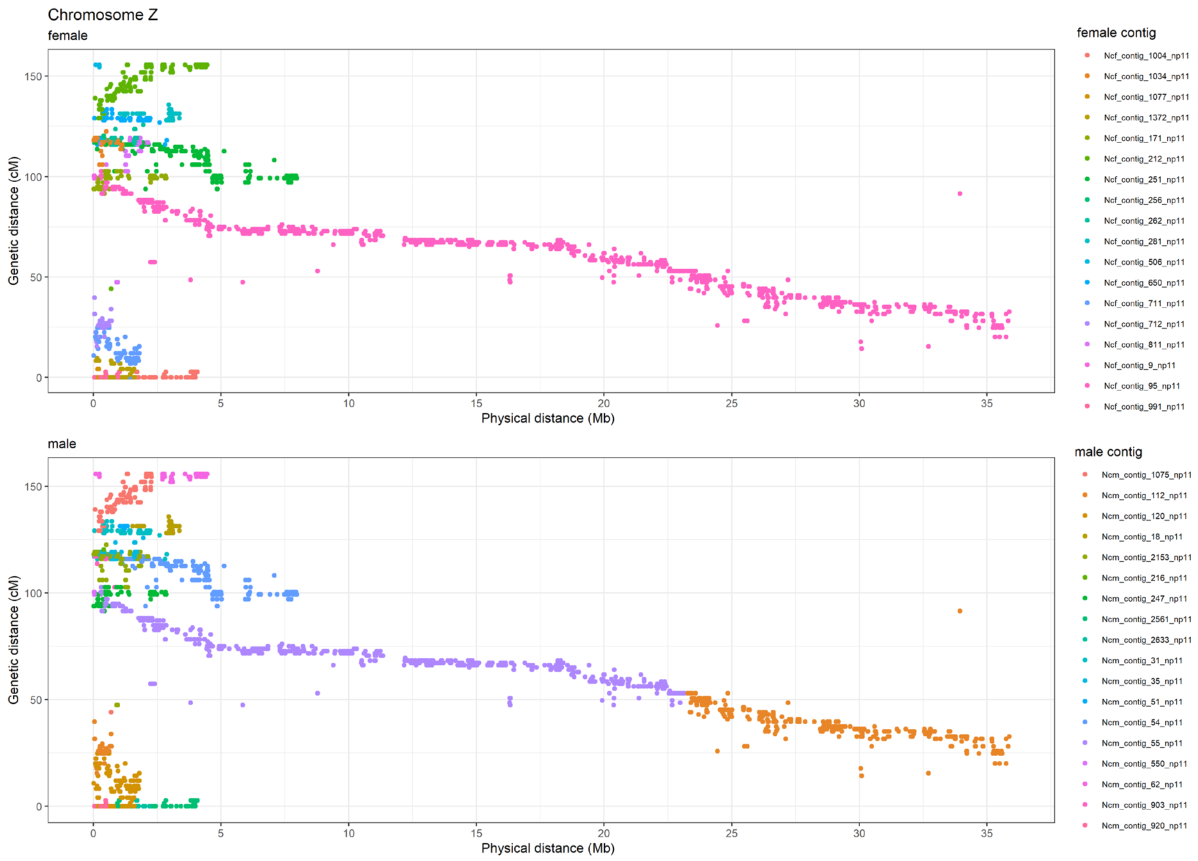

Figure S4. Linkage map genetic distances of the Z-linked contigs from the female reference genome (top) and the male genome assembly (bottom). Multiple contigs are stacked at physical positions close to 0 because positions are per contig and not for the chromosome.

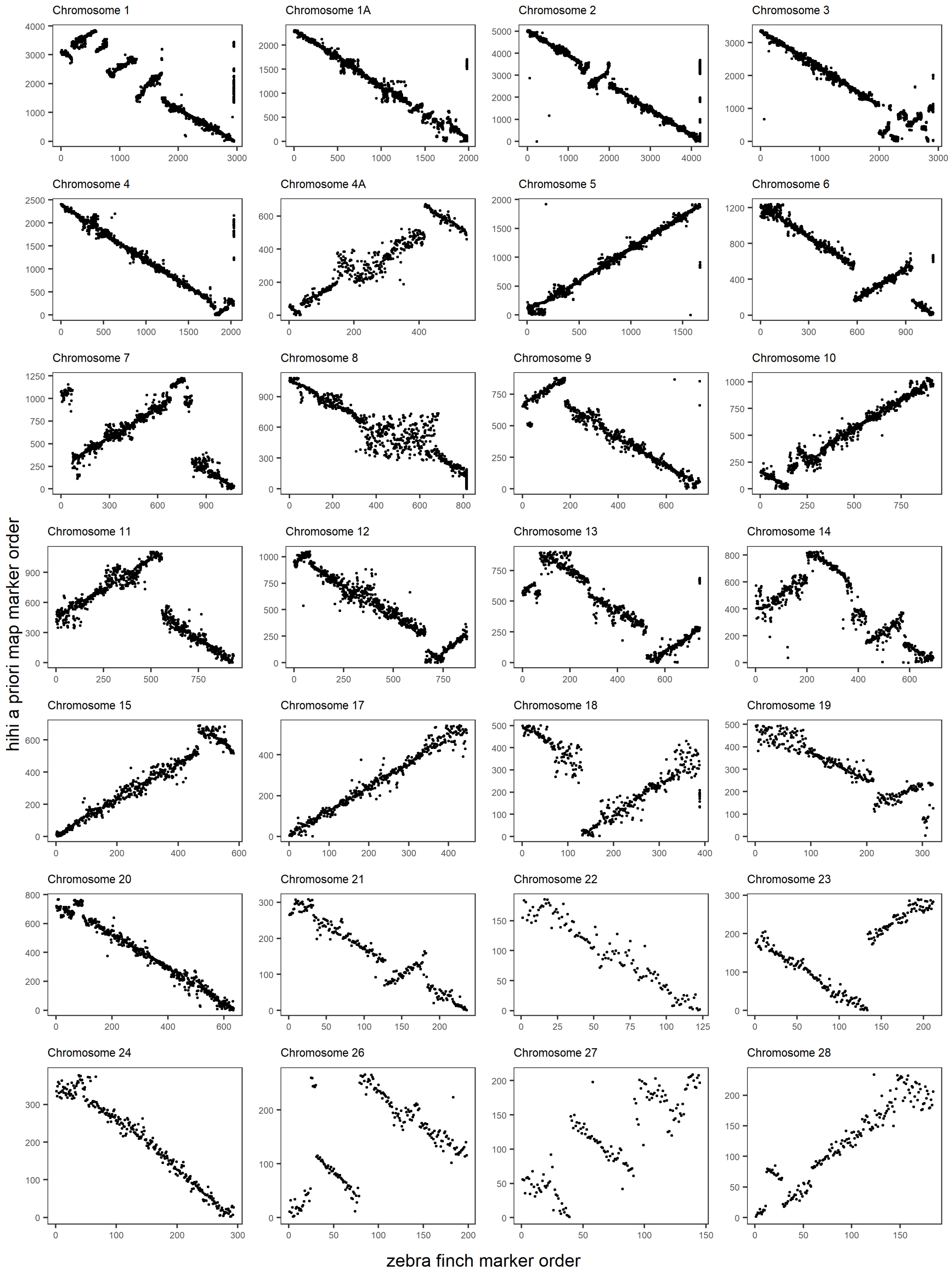

Figure S5. Hihi a priori map marker order, based on constructing linkage maps per-chromosome in the absence of physical information from the hihi genome, vs zebra finch marker order. A number of large blocks of discordant marker order are clear, and are likely due to an inability to resolve hihi marker order in regions of low recombination given the small number of individuals and high levels of linkage disequilibrium in the population.

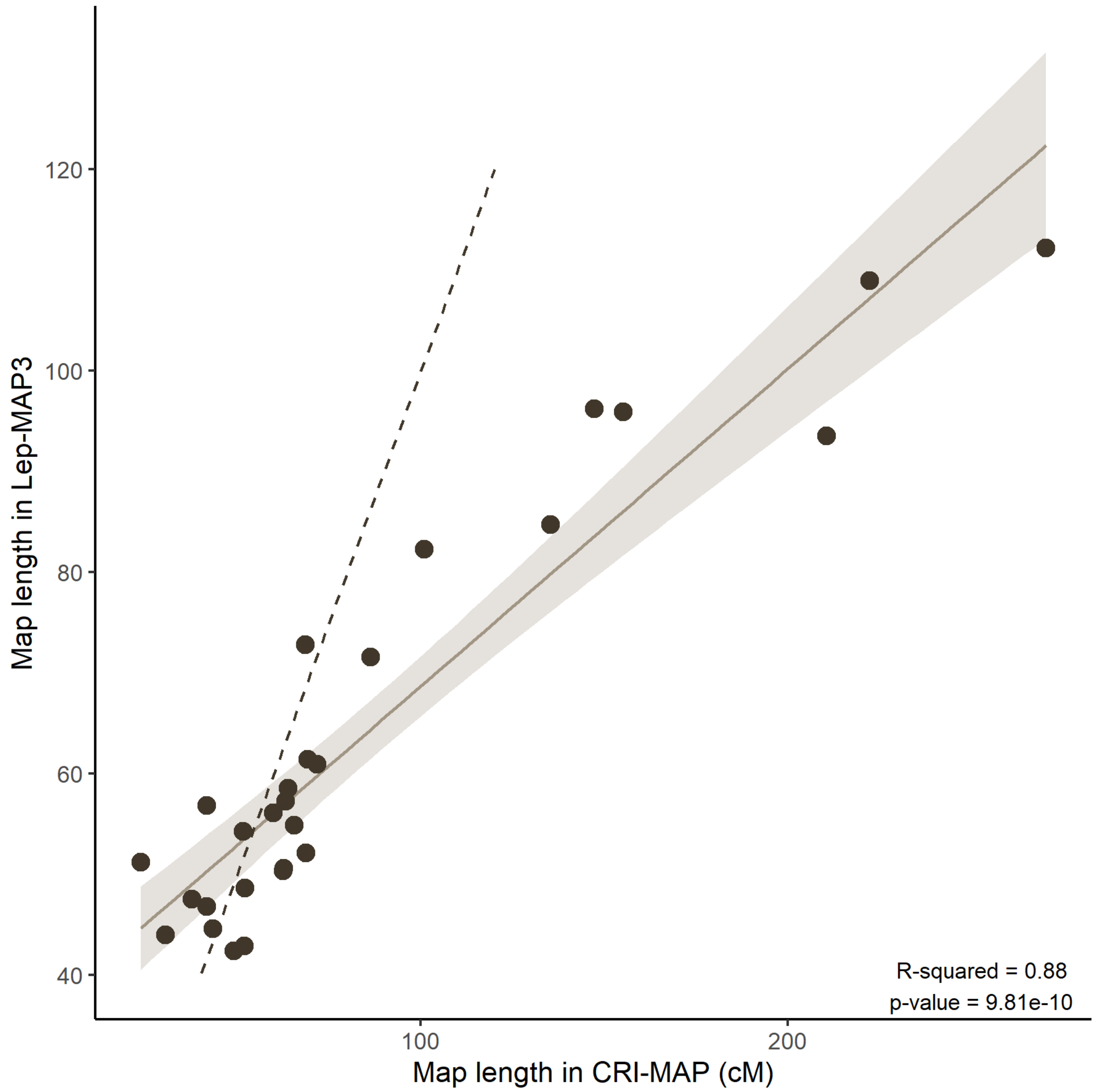

Figure S6. Comparison of map lengths for each chromosome using results from Lep-MAP3 (physical maps) and CRI-MAP. Solid brown line represents the linear regression and shaded area represents the 95% confidence interval. Dotted line represents a 1:1 relationship between map length in CRI-MAP and in Lep-MAP3.

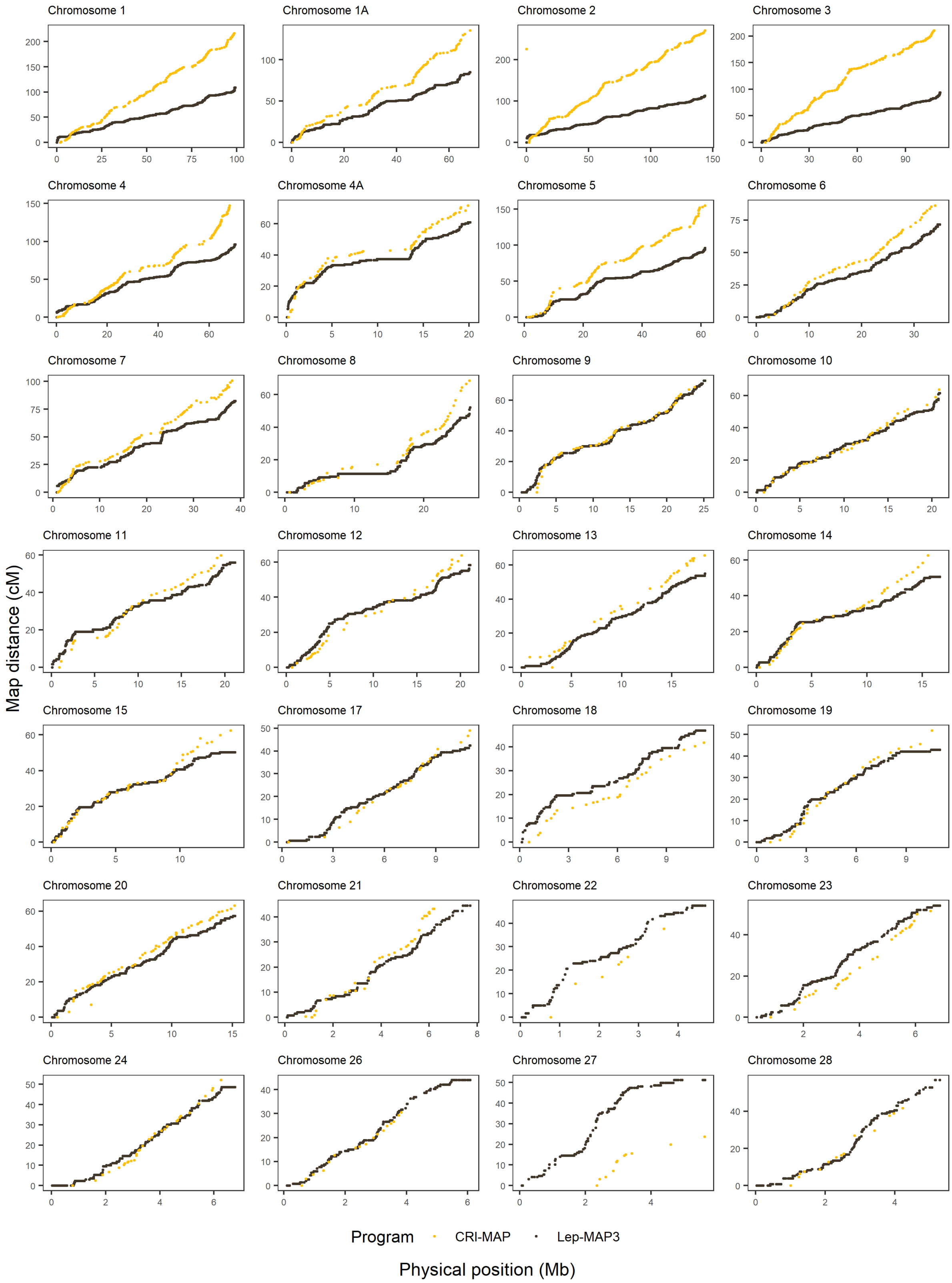

Figure S7. Recombination landscape of each chromosome as constructed by Lep-MAP3 (physical maps) and CRI-MAP respectively.

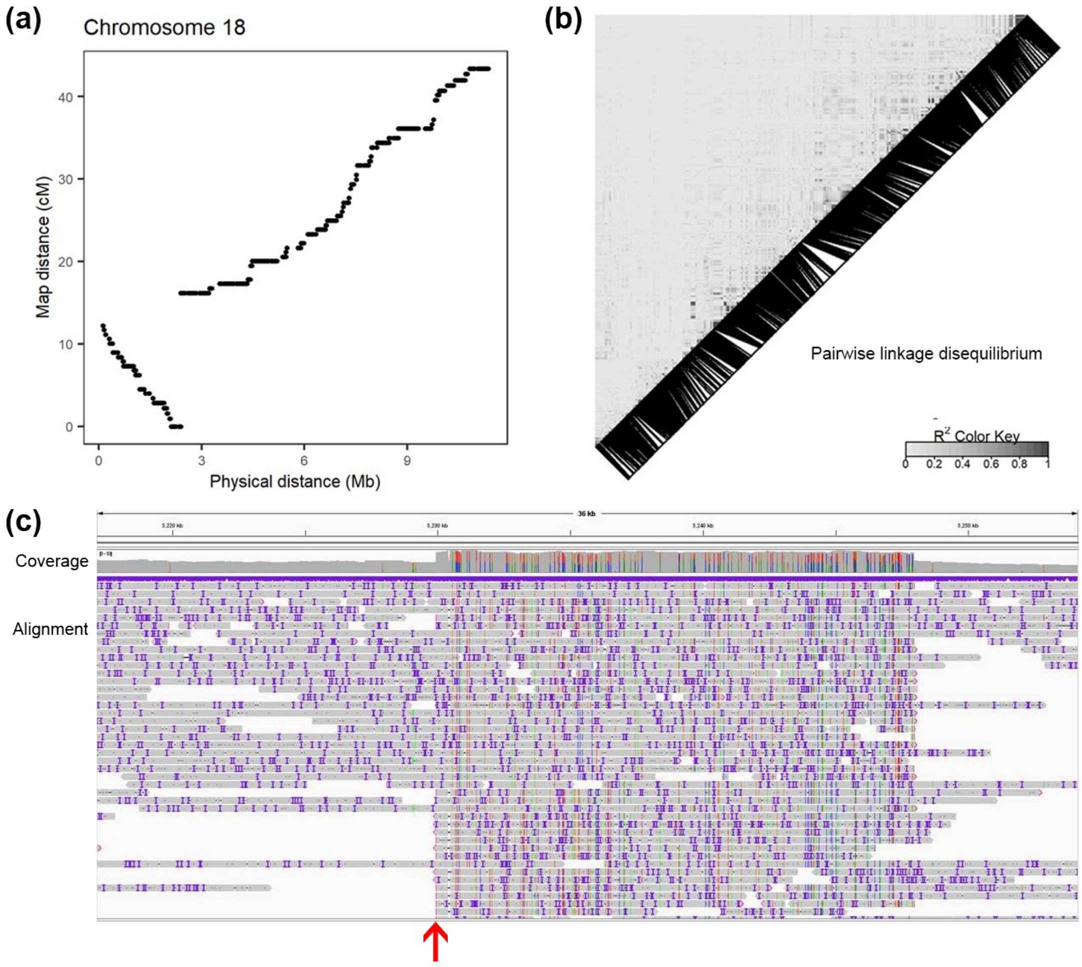

Figure S8. Evidence for an erroneous inversion in the assembly of chromosome 18 in the initial hihi reference genome. (a) Lep-MAP3 physical map for chromosome 18 constructed based on the physical ordering of markers in the hihi genome assembly. (b) Pairwise linkage disequilibrium between pairs of SNPs along chromosome 18. (c) Oxford Nanopore read coverage information across the misassembled region; high read coverage indicates that it is a highly repetitive region, which may explain the original misassembly; the likely misassembly point is indicated with a red arrow. The genome assembly was amended, and a new physical map built for chromosome 18.

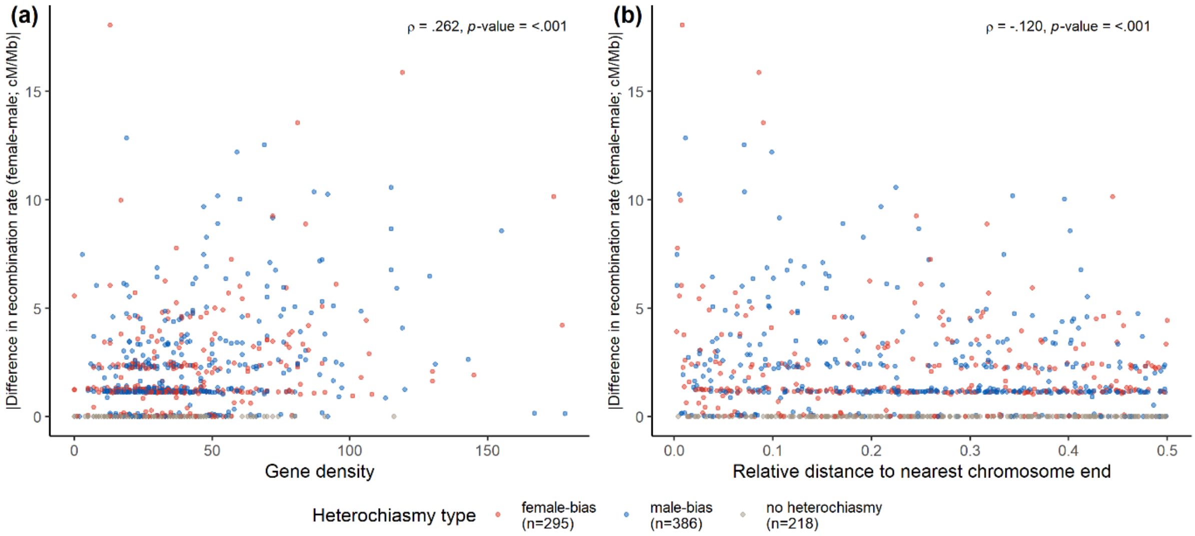

Figure S9. Relationship between absolute difference in recombination rate between sexes against (a) gene density and (b) relative distance to nearest chromosome end for all 1Mb intervals across the genome. The results of Spearman's Rho correlation tests are inset for each panel. The number of intervals in each category is provided in parentheses in the legend.

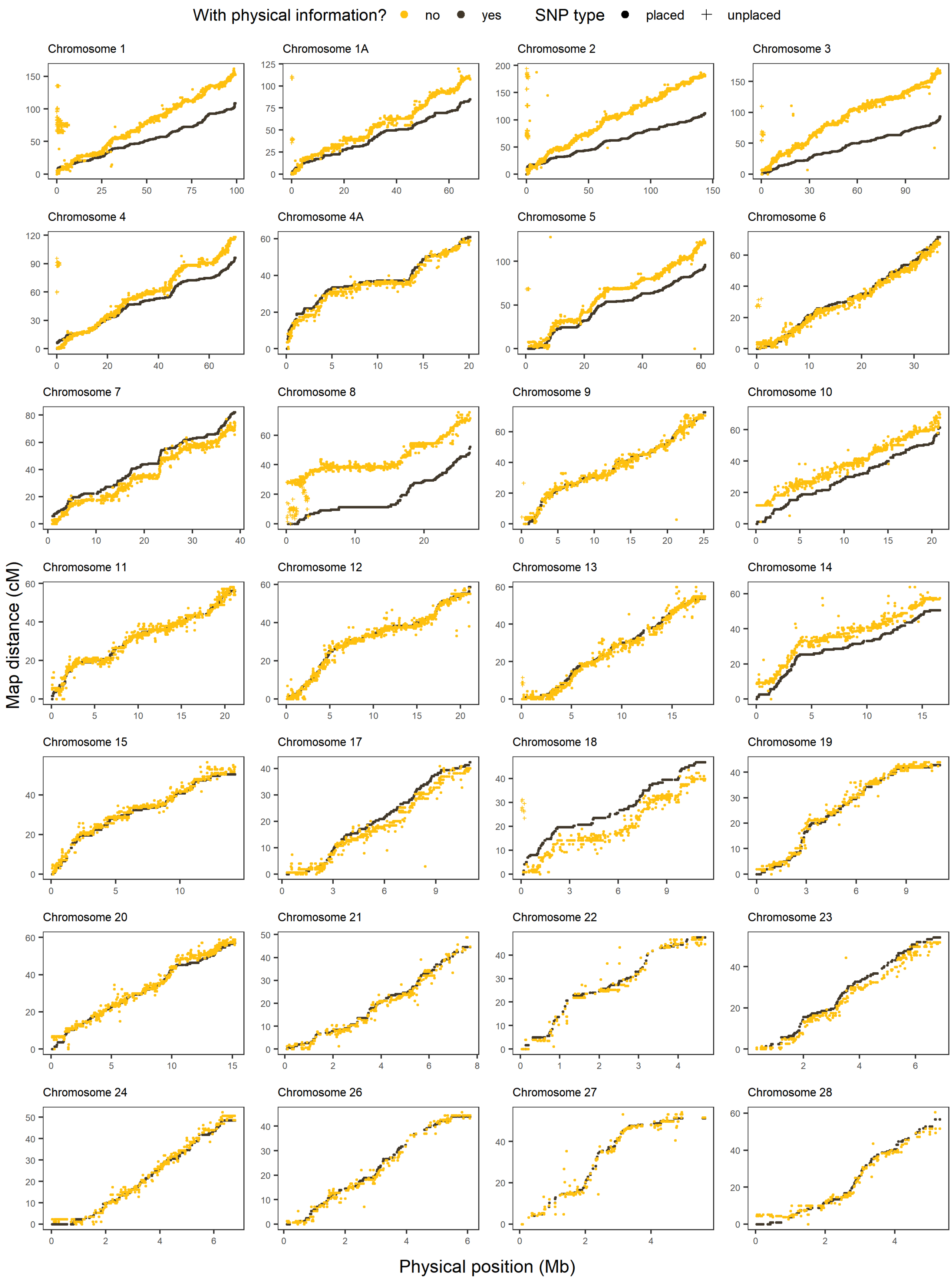

Figure S10. Recombination landscape of each chromosome as constructed by Lep-MAP3 with and without physical information respectively. Maps constructed without physical information include unplaced SNPs, which refer to SNPs that were mapped within contigs that did not meet the size criteria (>50kb) to be included in the published hihi genome assembly. The physical positions of these SNPs refer to their position within their respective contigs.

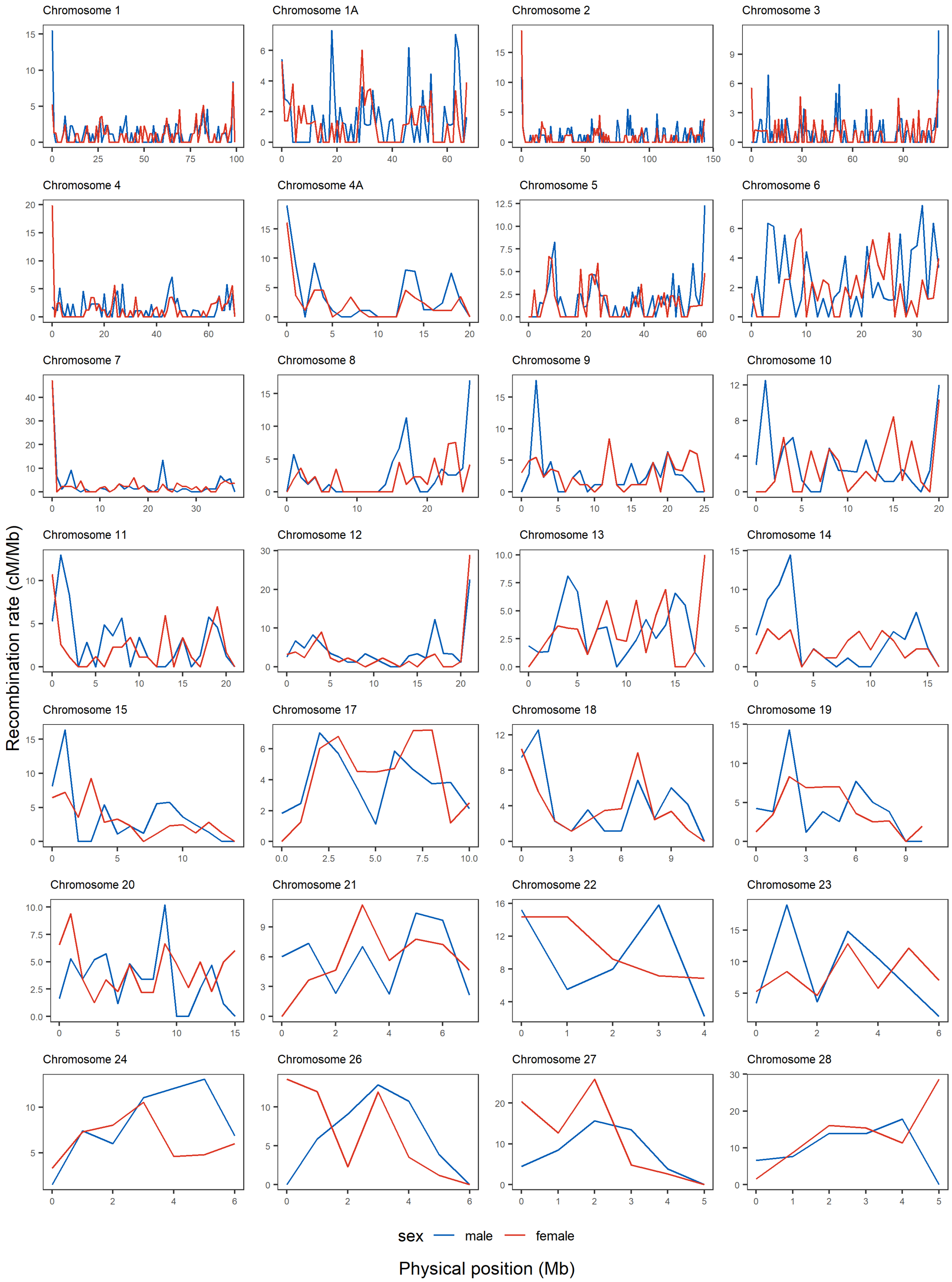

Figure S11. Male and female recombination rates per 1 Mb interval against physical position. Note the change in the x-axis scaling per chromosome to allow for visualisation.

Table S1. Results of zero-or-one inflated beta (ZOIB) regression modelling the relationship between the modulus of the rescaled heterochiasmy index and gene density, absolute and relative distance from chromosome ends for 1 Mb intervals across the genome. Bold values within each table indicate the credible intervals for the estimate of the effect of the term in the model; credible intervals that exclude zero are considered significant. An increase in gene density is associated with an increase in the modulus rescaled heterochiasmy index, while an increase in distance (absolute or relative) from chromosome ends is associated with a decrease.

**A. Number of genes (gene density)**							
	**Estimate**	**Est. Error**	**l-95% CI**	**u-95% CI**	**Rhat**	**Bulk_ESS**	**Tail_ESS**
**Intercept**	-1.49	0.15	-1.80	-1.20	1.00	2,465	2,544
**phi_Intercept**	-0.25	0.14	-0.53	0.04	1.00	2,525	2,551
**zoi_Intercept**	1.52	0.15	1.22	1.83	1.00	4,773	2,712
**coi_Intercept**	0.03	0.19	-0.33	0.40	1.00	4,921	2,943
**numGenes**	0.00	0.00	**0.00**	**0.01**	1.00	3,383	3,035
**phi_numGenes**	0.01	0.00	0.01	0.02	1.00	3,588	3,426
**zoi_numGenes**	-0.03	0.00	-0.04	-0.03	1.00	5,285	2,870
**coi_numGenes**	0.01	0.01	-0.00	0.02	1.00	3,342	2,982

**B. Distance to chromosome end**							
	**Estimate**	**Est. Error**	**l-95% CI**	**u-95% CI**	**Rhat**	**Bulk_ESS**	**Tail_ESS**
**Intercept**	-1.03	0.09	-1.20	-0.86	1.00	6,771	3,314
**phi_Intercept**	0.76	0.10	0.57	0.95	1.00	6,129	3,037
**zoi_Intercept**	-0.59	0.11	-0.80	-0.38	1.00	6,569	2,921
**coi_Intercept**	0.62	0.15	0.33	0.90	1.00	3,318	2,613
**distChrEnd**	-0.02	0.01	**-0.03**	**-0.01**	1.00	2,167	2,961
**phi_distChrEnd**	-0.03	0.01	-0.05	-0.02	1.00	2,249	3,335
**zoi_distChrEnd**	0.05	0.01	0.04	0.06	1.00	4,905	3,190
**coi_distChrEnd**	-0.02	0.01	-0.03	-0.01	1.00	4,426	2,838

**C. Relative distance to chromosome end**							
	**Estimate**	**Est. Error**	**l-95% CI**	**u-95% CI**	**Rhat**	**Bulk_ESS**	**Tail_ESS**
**Intercept**	-1.00	0.13	-1.25	-0.75	1.00	5,674	3,402
**phi_Intercept**	0.76	0.13	0.49	1.02	1.00	5,731	3,285
**zoi_Intercept**	-0.08	0.13	-0.34	0.18	1.00	7,074	2,916
**coi_Intercept**	0.34	0.19	-0.01	0.71	1.00	6,933	3,237
**relDistChrEnd**	-0.60	0.25	**-1.09**	**-0.11**	1.00	4,087	3,362
**phi_relDistChrEnd**	-0.98	0.25	-1.46	-0.50	1.00	4,114	3,091
**zoi_relDistChrEnd**	0.62	0.23	0.17	1.09	1.00	7,190	2,965
**coi_relDistChrEnd**	-0.16	0.31	-0.78	0.45	1.00	6,852	2,848

Table S2. Results of gene overrepresentation test in the target intervals with and without false discovery rate (FDR) correction.

Annotation dataset	GO aspect	Number of significant GO terms	
		FDR	No correction
Complete GO	GO biological process complete	0	150
	GO cellular component complete	2	38
	GO molecular function complete	0	53
PANTHER GO-slim	Biological Process	0	57
	Cellular Component	0	16
	Molecular Function	0	13

Table S3. GO terms significantly enriched in regions of extreme heterochiasmy

GO term	Ontology	Description	Fold Enrichment	p-value	FDR
GO:0005882	Cellular	intermediate filament	4.33	2.79E-05	4.08E-02
GO:0045111	Cellular	intermediate filament cytoskeleton	3.82	4.88E-05	3.57E-02

Table S4. Differences in calculation and interpretation of measures of heterochiasmy.

Source	Formula (name)	Interpretation
Hansson et al., 2005	$\frac{\text{female map length (cM)}}{\text{male map length (cM)}}$	Ratio of female to male linkage map lengths
Lovich and Gibbons, 1992; Poissant et al., 2010; van Oers et al., 2014	$\frac{\text{map length of sex with longer map (cM)} - \text{map length of sex with shorter map (cM)}}{\text{map length of sex with shorter map (cM)}}$ (size dimorphism index)	Describes differences in map lengths between sexes relative to the sex with the shorter map
Mank, 2009; Malinovskaya et al., 2020	$\frac{\text{female map length (cM)} - \text{male map length (cM)}}{\text{average map length (cM)}}$ (heterochiasmy index)	Describes differences in map lengths between sexes relative to the sex-averaged map
-	$\text{female map length (cM)} - \text{male map length (cM)}$	Differences in raw values of recombination

References

Hansson B, Åkesson M, Slate J, Pemberton JM (2005) Linkage mapping reveals sex-dimorphic map distances in a passerine bird. Proc R Soc B Biol Sci 272:2289–2298

Lovich JE, Gibbons JW (1992) A review of techniques for quantifying sexual size dimorphism. Growth, Dev Aging 56:269–281

Malinovskaya LP, Tishakova K, Shnaider EP, Borodin PM, Torgasheva AA (2020) Heterochiasmy and sexual dimorphism: The case of the barn swallow (*Hirundo rustica*, Hirundinidae, Aves). Genes (Basel) 11:1–11

Mank JE (2009) The evolution of heterochiasmy: The role of sexual selection and sperm competition in determining sex-specific recombination rates in eutherian mammals. Genet Res (Camb) 91:355–363

van Oers K, Santure AW, De Cauwer I, Van Bers NEM, Crooijmans RPMA, Sheldon BC et al. (2014) Replicated high-density genetic maps of two great tit populations reveal fine-scale genomic departures from sex-equal recombination rates. Heredity (Edinb) 112:307–316

Poissant J, Hogg JT, Davis CS, Miller JM, Maddox JF, Coltman DW (2010) Genetic linkage map of a wild genome: Genomic structure, recombination and sexual dimorphism in bighorn sheep. BMC Genomics 11:524
